# GFP fluorescence tagging alters dynamin-related protein 1 oligomerization dynamics and creates disassembly-refractory puncta to mediate mitochondrial fission

**DOI:** 10.1038/s41598-020-71655-x

**Published:** 2020-09-08

**Authors:** Felipe Montecinos-Franjola, Brianna L. Bauer, Jason A. Mears, Rajesh Ramachandran

**Affiliations:** 1 Department of Physiology and Biophysics, Case Western Reserve University School of Medicine, Cleveland, OH 44106 USA; 2 Department of Pharmacology, Case Western Reserve University School of Medicine, Cleveland, OH 44106 USA; 3 Center for Mitochondrial Diseases, Case Western Reserve University School of Medicine, Cleveland, OH 44106 USA; 4 Cleveland Center for Membrane and Structural Biology, Case Western Reserve University School of Medicine, Cleveland, OH 44106 USA

**Keywords:** Biochemistry, Biophysics, Biological techniques, Optical spectroscopy

## Abstract

Green fluorescent protein (GFP)-tagging is the prevalent strategy to monitor protein dynamics in living cells. However, the consequences of appending the bulky GFP moiety to the protein of interest are rarely investigated. Here, using a powerful combination of quantitative fluorescence spectroscopic and imaging techniques, we have examined the oligomerization dynamics of the GFP-tagged mitochondrial fission GTPase dynamin-related protein 1 (Drp1) both in vitro and in vivo. We find that GFP-tagged Drp1 exhibits impaired oligomerization equilibria in solution that corresponds to a greatly diminished cooperative GTPase activity in comparison to native Drp1*.* Consequently, GFP-tagged Drp1 constitutes aberrantly stable, GTP-resistant supramolecular assemblies both in vitro and in vivo, neither of which reflects a more dynamic native Drp1 oligomerization state. Indeed, GFP-tagged Drp1 is detected more frequently per unit length over mitochondria in *Drp1-null* mouse embryonic fibroblasts (MEFs) compared to wild-type (*wt*) MEFs, indicating that the drastically reduced GTP turnover restricts oligomer disassembly from the mitochondrial surface relative to mixed oligomers comprising native and GFP-tagged Drp1. Yet, GFP-tagged Drp1 retains the capacity to mediate membrane constriction in vitro and mitochondrial division in vivo. These findings suggest that instead of robust assembly-disassembly dynamics, persistent Drp1 higher-order oligomerization over membranes is sufficient for mitochondrial fission.

## Introduction

Fluorescence labeling with genetically encoded variants of GFP is the expedient method for the real-time monitoring of protein distribution and dynamics in living cells^1–3^. However, seldom dissected are the plausible negative structural and functional ramifications of attaching the sizeable, ~ 27 kDa GFP moiety to the protein of interest. Indeed, in many instances, GFP-tagging has been shown to generate unstable fusion products^4,5^, cause aberrant protein localization^6^, promote aggregation^7^, prevent assembly^8^, and/or perturb protein function in more than subtle ways^9–12^. Such scrutiny has never been imposed on GFP-tagged dynamin-related protein 1 (Drp1), a long-studied yet controversial mechanoenzymatic GTPase recruited from the cytosol to the mitochondrial surface to mediate mitochondrial division^13,14^.

Drp1 self-assembles into an organelle-enwrapping helical oligomer over premarked division sites on the mitochondrial outer membrane (MOM)^15,16^. GTP-driven conformational rearrangements in the membrane-bound Drp1 oligomer coupled to dynamic assembly-disassembly cycles over narrow, tubular membrane intermediates seemingly catalyzes mitochondrial constriction for fission^17,18^. However, discrepancies abound on the oligomerization state(s) of Drp1 in the cytosol^19–21^, the identity of the oligomeric species selectively recruited to mitochondria^22–24^, and the role of GTP-driven conformational dynamics and assembly-disassembly cycles in the fission process^25^. GFP-tagged Drp1, used primarily as a surrogate in cellular studies, has yielded conflicting results in comparison to native or native-like His-tagged Drp1, both in vitro and in vivo. Untagged and His-tagged Drp1 exist in comparable, dynamic, dimer-tetramer-higher-order-oligomer equilibria in solution in vitro^21,22,26^. GFP-tagged Drp1, by contrast, resides primarily as a tetramer in the cytosol in vivo^19,27,28^. Likewise, Drp1 dimers, but not oligomers, preferentially associate with the MOM-anchored Drp1 receptor mitochondrial fission factor (Mff) in vitro^22,29^. On the other hand, Mff selectively recruits higher-order GFP-tagged Drp1 oligomers, but not dimers, in vivo^23,24^. More recently, GFP-tagged Drp1 was shown to constrict as well as mediate the fission of highly curved membrane tubes upon GTP hydrolysis in vitro^30^. Yet, only membrane tube constriction, but not fission, was previously observed with both untagged and His-tagged Drp1^17,18,31^. Similar discrepancies persist in the case of small organic dye-labeled Drp1. Surface Cys-modified, Alexa Fluor 488 (AF488)-labeled Drp1 preferentially bound and stabilized pre-curved membrane tubes in vitro, yet was incapable of further membrane constriction in the presence of GTP in vitro^32,33^. Conversely, similarly modified BODIPY-FL (BODIPY)-labeled Drp1 efficiently catalyzed Drp1-*wt*-like constriction of relatively flat membranes, but not fission, under comparable conditions^17^. These conflicting results suggested that the various fluorescent tags adorning Drp1 might not be passive constituents as originally assumed but instead actively influence Drp1 structure and/or function.

Here we have used fluorescence correlation spectroscopy (FCS), raster-image correlation spectroscopy (RICS), and number and brightness (N&B) analysis, in combination with live-cell confocal imaging to directly compare the different fluorescently labeled Drp1 constructs currently employed, and reveal their disparities. We show that GFP-tagging at either end disrupts Drp1 oligomerization equilibria both in solution and on membranes by favoring Drp1 higher-order self-assembly and limiting GTP hydrolysis-mediated oligomer disassembly. Modification by the bulky and charged AF488 dye selectively impairs assembly-stimulated Drp1 GTPase activity, whereas the smaller but hydrophobic BODIPY dye elicits Drp1 aggregation in solution upon prolonged incubation. Regardless, GFP-tagged Drp1 constricts membrane tubes in vitro and divides mitochondria in vivo suggesting that persistent Drp1 self-assembly on membranes is sufficient for mitochondrial membrane fission. Importantly, our results caution against the interpretation of results from disparately labeled Drp1 constructs without appropriate controls, and biochemical and biophysical information in place.

## Results

### GFP-tagging impairs Drp1 oligomerization propensity and dynamics

**Figure 1:**
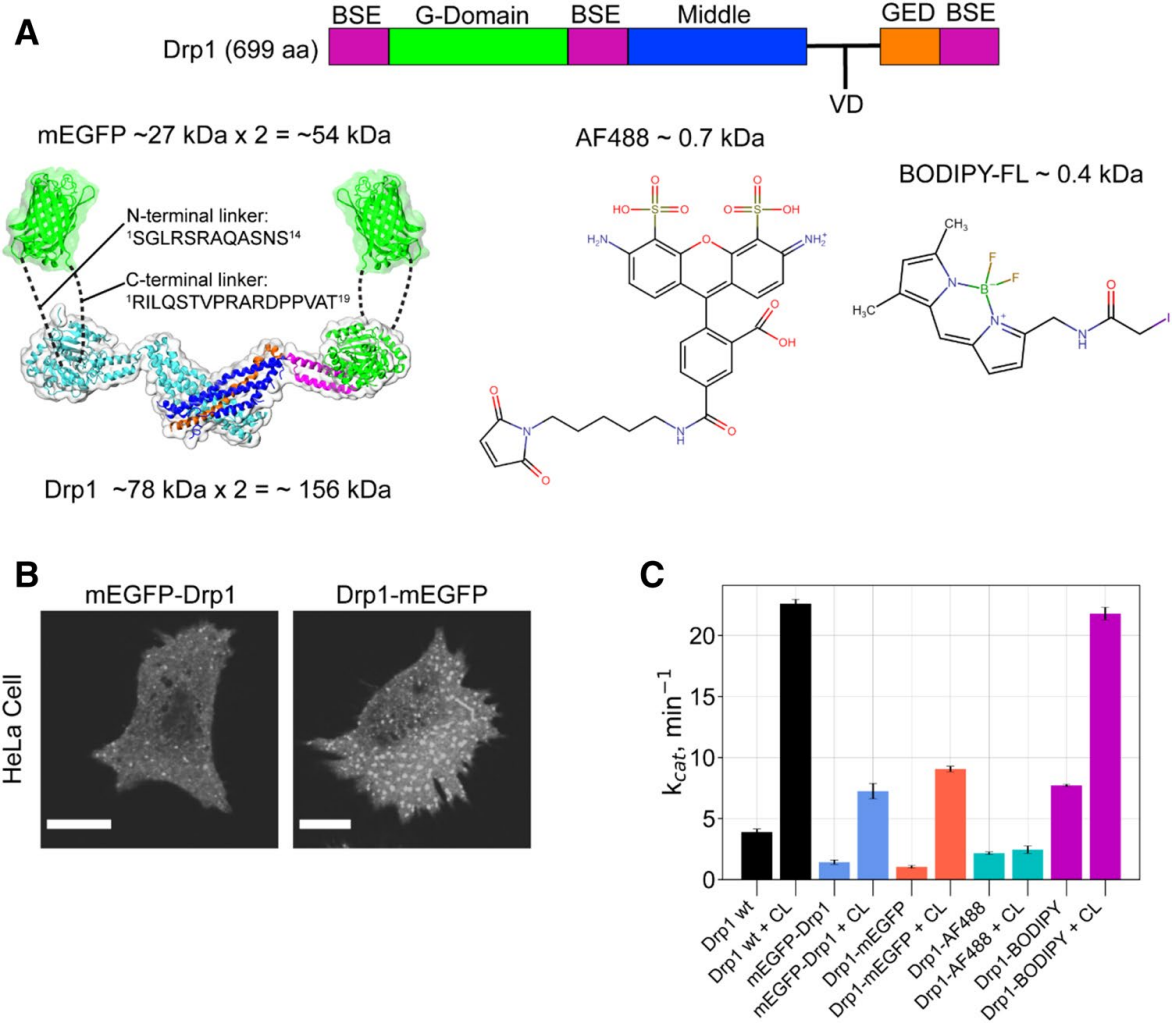
GFP-tagging differentially influences Drp1 activity both in vitro and in vivo. (**A**) (*left*) Drp1 domain arrangement, and 3D structures of the minimal Drp1 dimer (PDB ID: 4BEJ) and mEGFP (PDB ID: 2Y0G) drawn to scale. A Drp1 monomer is color-coded in correspondence with the above domain arrangement. The sequences of the linkers connecting mEGFP to Drp1 at the N- and C-termini are shown and are illustrated by dashed lines. *G* GTPase domain; *BSE* bundle signaling element; *VD* variable domain; and *GED* GTPase effector domain. The structural models were prepared using VMD v1.9.3^34^ available at https://www.ks.uiuc.edu/Research/vmd/. (right) Chemical structures of AF488 C5-maleimide and BODIPY-FL C1-iodoacetamide used for Drp1 surface-Cys fluorescence labeling. The chemical structures were prepared using MolView v2.4 available at https://molview.org/. (**B**) Confocal fluorescence images (shown in grayscale) of representative HeLa cells overexpressing either mEGFP-Drp1 (left) or Drp1-mEGFP (right). Numerous large cytosolic aggregates were observed for Drp1-mEGFP relative to mEGFP-Drp1. Images were prepared using the software platform Fiji^35^. Scale bars, 10 μm. (**C**) Basal (protein alone) and CL-stimulated GTPase activities of the various fluorescently labeled Drp1 constructs relative to Drp1-wt (0.5 μM protein final; 150 μM total lipids final). Calculated GTP turnover numbers (k_cat_, min^-1^) are an average of three experiments ± S.D. Bar plots were prepared using matplotlib v3.2.2 available at https://matplotlib.org/3.2.2/api/_as_gen/matplotlib.pyplot.boxplot.html.

We fused monomeric enhanced GFP (mEGFP) to the N- or C-terminus of the ubiquitously expressed Drp1 isoform 3 (699 aa) (Fig. 1A). N-terminally tagged mEGFP-Drp1 and C-terminally tagged Drp1-mEGFP were overexpressed in *E. coli* for recombinant protein production and biochemical and biophysical characterization in vitro, and in MEFs for examining Drp1 quaternary structure, oligomerization dynamics, and function in vivo. Linker sequences connecting Drp1 to mEGFP were kept identical between the corresponding *E. coli* and mammalian expression constructs.

The discontinuous, multi-domain architecture of the Drp1 molecule positions its N- and C-termini in close proximity, and near the GTPase (G) domain (Fig. 1A)^36^. Hence, it is assumed that GFP-tagging at either end results in a similar 3D fold and stability for the two different Drp1 fusion constructs. Oddly, however, in comparison to mEGFP-Drp1, Drp1-mEGFP expressed rather poorly in *E. coli,* had limited solubility, and yielded relatively less stable protein (data not shown). Consistent with this observation, we frequently noticed a large-scale aggregation of overexpressed Drp1-mEGFP relative to mEGFP-Drp1 in the cytosol of transfected HeLa cells (Fig. 1B). We could, therefore, use Drp1-mEGFP only for a select number of comparative experiments with mEGFP-Drp1 in vitro.

**Figure 2:**
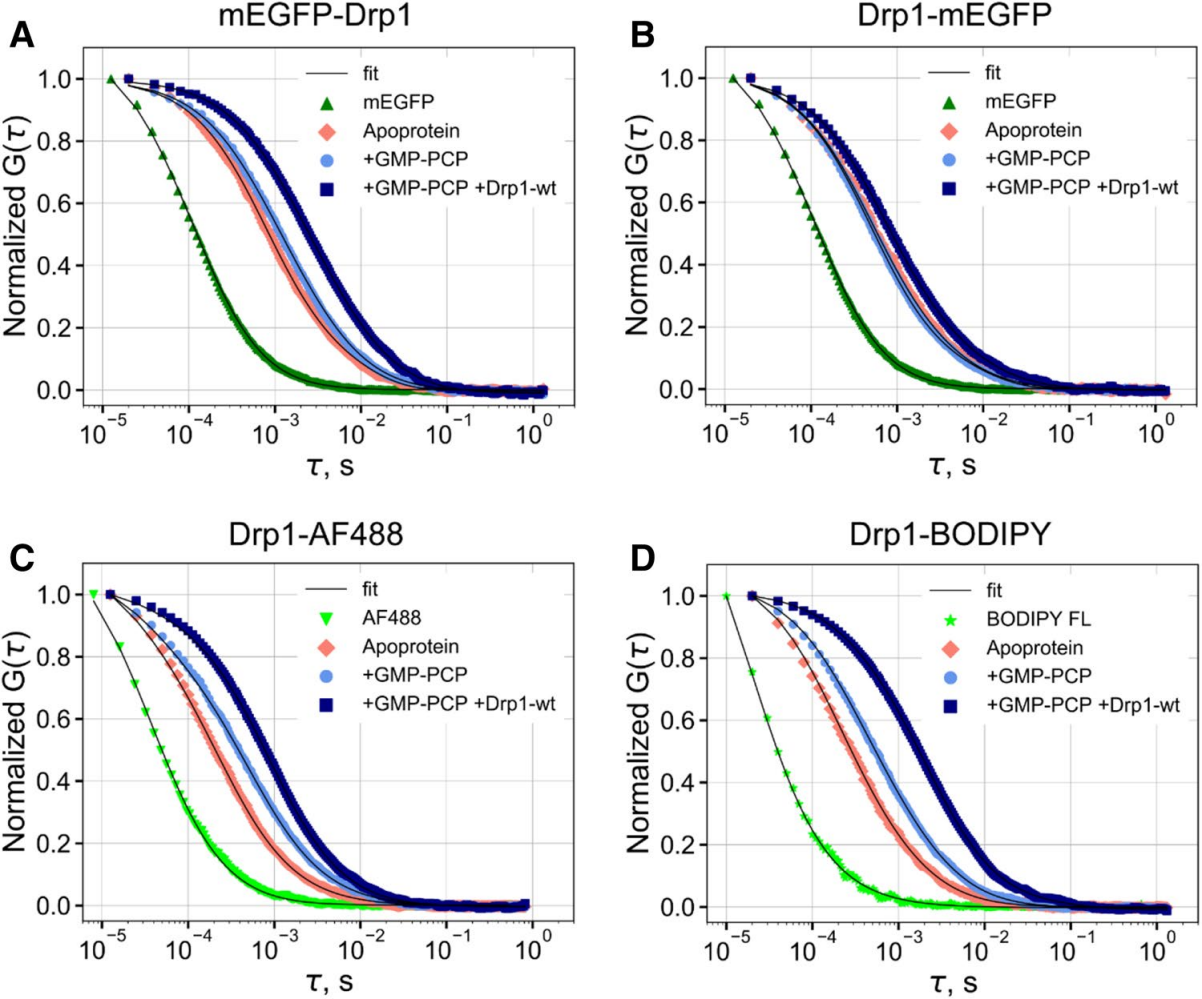
FCS measurements reveal impaired oligomerization dynamics for GFP-tagged Drp1 in vitro. (**A**–**D)** Autocorrelation curves for the various fluorescently labeled Drp1 constructs in the absence and presence of the indicated additives obtained using single-point FCS. In each case, the corresponding fluorescent label alone (mEGFP or small organic dye) was also measured. The recovered ‘D’ from the fitting of the data (solid lines) to one or two species are shown in Table 1. The data and fits were normalized to facilitate direct comparisons. Apoprotein refers to the indicated fluorescently labeled Drp1 construct in the absence of nucleotide. Autocorrelation plots were prepared using matplotlib v3.2.2 available at https://matplotlib.org/3.2.2/api/_as_gen/matplotlib.pyplot.boxplot.html.

Remarkably, mEGFP-Drp1 and Drp1-mEGFP exhibited a similar > 50% reduction in GTP turnover relative to Drp1-*wt* under both basal and cardiolipin (CL)-stimulated conditions^21^ (Fig. 1C). These data indicated that the GFP moiety regardless of its location with respect to the Drp1 termini posed a steric hindrance to either nucleotide-dependent Drp1 helical self-assembly and/or inter-subunit G domain dimerization^31,37^ essential for cooperative GTP hydrolysis both in solution and on membranes^31^. To test this possibility, we employed single-point FCS^38–41^, a microscopy-based biophysical technique that measures the size of a fluorescent particle through the determination of its diffusion coefficient, D, based on the temporal autocorrelation of fluorescence intensity fluctuations within a defined focal volume (typically ~ 1 fL; see Methods for a detailed description). We first measured the diffusion of GFP-tagged Drp1 at physiologically relevant 0.5 μM concentration in solution^26^, in the absence and presence of the non-hydrolyzable GTP analogue, GMP-PCP (Fig. 2A,B). GTP binding promotes Drp1 higher-order self-assembly, which in turn stimulates cooperative GTP hydrolysis and oligomer disassembly^18,42^. In the absence of GMP-PCP, by comparison with purified mEGFP alone, mEGFP-Drp1 and Drp1-mEGFP both exhibited significantly slower diffusion (D_1_) consistent with their relatively large size and elongated dimensions. GMP-PCP addition elicited a further quantifiable reduction in the diffusion of both mEGFP-Drp1 and Drp1-mEGFP as determined by the appearance of a slow-moving, minor diffusional component (D_2_) in the autocorrelation curve indicative of some degree of higher-order self-assembly (Table 1). However, no major reduction was observed for the fast-moving, major component (D_1_) in either case and particularly for Drp1-mEGFP, suggesting that GFP-tagged Drp1, regardless of GFP end, is poorly responsive to GTP binding (Table 1). By contrast, Drp1-AF488 and Drp1-BODIPY both exhibited a substantial, GMP-PCP-dependent reduction in D_1_, which was pronouncedly greater for Drp1-BODIPY, indicative of a robust, GTP binding-dependent higher-order self-assembly (Fig. 2C,D, Table 1).

**Table 1:** Diffusion coefficients (D) in solution determined using FCS.

Protein	D_1_, μm^2^/s	D_2_, μm^2^/s
mEGFP	98.3 ± 2.9 (1.0)	ND
**mEGFP-Drp1**		
apoprotein	28.1 ± 3.5 (1.0)	ND
+ 1 mM GMP-PCP	25.6 ± 0.5 (0.7)	6.8 ± 2.1
+ 1 mM GMP-PCP + 1 mM Drp1-wt	19.0 ± 1.5 (0.6)	4.1 ± 0.5
**Drp1-mEGFP**		
apoprotein	25.2 ± 0.5 (1.0)	ND
+ 1 mM GMP-PCP	31.7 ± 2.8 (0.8)	3.8 ± 2.2
+ 1 mM GMP-PCP + 1 mM Drp1-wt	20.4 ± 0.9 (0.8)	1.4 ± 0.7
**Drp1-AF488**		
apoprotein	34.5 ± 1.1 (0.6)	ND
+ 1 mM GMP-PCP	18.9 ± 0.8 (0.7)	ND
+ 1 mM GMP-PCP + 1 mM Drp1-wt	14.4 ± 0.7 (0.9)	ND
**Drp1-BODIPY FL**		
Apoprotein	32.4 ± 0.8 (0.7)	ND
+ 1 mM GMP-PCP	18.5 ± 1.2 (0.8)	ND
+ 1 mM GMP-PCP + 1 mM Drp1-wt	6.8 ± 0.9 (0.9)	ND

Fluorescently labeled proteins were analyzed at 0.5 μM final concentration.

GMP-PCP (1 mM final) and unlabeled Drp1-wt (1 μM final) were added to the pre-equilibrated solutions.

The uncertainties are the standard deviations (S.D.) of three measurements.

The fractional amplitudes of the autocorrelation function for each component is shown in parenthesis.

ND, not determined.

The FCS data of dye-labeled Drp1 was fit using a fixed fast component to account for trace amounts of the free dye (D_AF488_ = 435 μm^2^/s, D_BODIPY_ = 476 μm^2^/s).

Likewise, the addition of a 2X molar excess of unlabeled Drp1-*wt* to the reaction to potentiate concentration-dependent Drp1 higher-order self-assembly elicited only a modest response from GFP-tagged Drp1 relative to dye-labeled Drp1 (Table 1). The latter robustly co-polymerized with Drp1-*wt* as evidenced by a marked reduction in D_1_. Interestingly, however, unlike Drp1-BODIPY, which showed *wt*-like CL-stimulated GTPase activity (Fig. 1C), Drp1-AF488 had very little GTPase activity under both basal and CL-stimulated conditions (Fig. 1C), indicating that the AF488 moiety selectively impaired GTP hydrolysis-dependent Drp1 oligomer disassembly likely essential for continuous cycles of GTP binding and hydrolysis. Drp1-BODIPY, by contrast, showed a greater basal GTPase activity than Drp1-*wt* indicating a spontaneous, time-dependent higher-order aggregation attributable to the small hydrophobic dye moiety upon prolonged incubation in solution (Fig. 1C). Nonetheless, these data revealed that GFP-tagging potently impairs Drp1 oligomerization propensity as well as nucleotide-dependent dynamics.

### GFP-tagging alters Drp1 oligomerization state and equilibria

**Figure 3:**
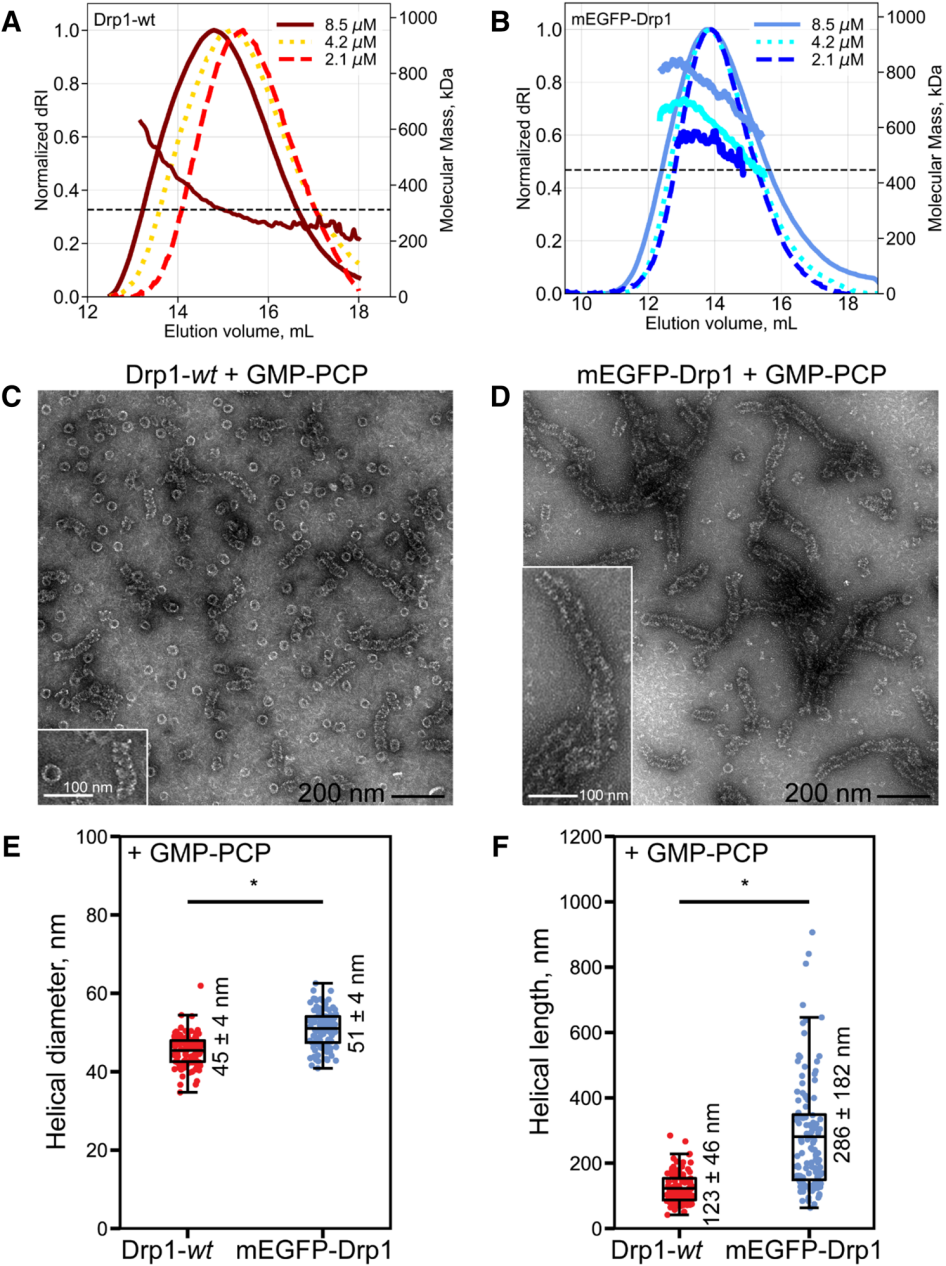
SEC-MALS and EM reveal altered quaternary structure and higher-order oligomerization states for mEGFP-Drp1 in vitro. **(A**,**B)** SEC-MALS profiles of Drp1-wt (**A**) and mEGFP-Drp1 (**B**) as a function of total loaded protein concentration before sieving. The horizontal dashed line in each plot indicates the approximate molecular mass of a corresponding tetramer (~ 328 kDa for Drp1-*wt* and ~ 444 kDa for mEGFP-Drp1). For Drp1-*wt*, molar mass distribution for only 8.5 μM loading protein concentration is shown. Drp1-wt displayed elution profile shifts as a function of protein concentration typically observed for self-associating systems in dynamic equilibrium. mEGFP-Drp1 showed no such apparent elution profile shifts and primarily sampled tetramer or higher oligomeric states. **(C**,**D)** Representative negative-stain EM images of Drp1-*wt* (**C**) and mEGFP-Drp1 (**D**) in the presence of GMP-PCP. Scale bar, 200 nm. Insets show magnified images of representative self-assembled higher-order structures. Inset scale bar, 100 nm. (**E**,**F**) Boxplots of helical diameter (**E**) and lengths (**F**) comparing Drp1-*wt* and mEGFP-Drp1 in the presence of GMP-PCP. Statistically significant differences between groups are indicated by a star. Mean ± S.D. is indicated next to each box. For helical diameters in panel E, Drp1-*wt* n = 114; mEGFP-Drp1 n = 105, p < 0.0001. For helical lengths in panel F, Drp1-*wt* n = 116; mEGFP-Drp1 n = 117, p < 0.0001. SEC-MALS profiles and box and whisker plots were prepared using matplotlib v3.2.2 available at https://matplotlib.org/3.2.2/api/_as_gen/matplotlib.pyplot.boxplot.html.

Even after taking into account the larger molecular mass and the disparate hydrodynamic volume (Supporting Fig. S1), we noted a significantly slower diffusional rate for GFP-tagged Drp1 compared to dye-labeled Drp1 under steady-state conditions (Table 1). These data indicated a larger oligomerization state for GFP-tagged Drp1 relative to Drp1-*wt* even at physiologically relevant low protein concentrations in solution. We used size-exclusion chromatography coupled to multi-angle light scattering (SEC-MALS) to determine the oligomerization state(s) of mEGFP-Drp1 relative to Drp1-*wt* in solution, as well as any concentration-dependent changes in oligomerization equilibria (Fig. 3A-B). Due to its poor solubility and yield, SEC-MALS was not feasible with Drp1-mEGFP. As previously observed^21^, Drp1-*wt* displayed dynamic oligomerization equilibria as evidenced by pronounced concentration-dependent shifts in SEC elution profiles (Fig. 3A). Further, the MALS data revealed that the oligomerization states ranged from minimal dimers to higher-order oligomers under these conditions (Fig. 3A). In stark contrast, mEGFP-Drp1 primarily constituted tetramers and higher-order oligomers, with no semblance of dimers (Fig. 3B). Moreover, mEGFP-Drp1 displayed significantly poor oligomerization dynamics as demonstrated by the lack of similar concentration-dependent elution peak shifts (Fig. 3B). These data indicated that GFP-tagging significantly disrupts Drp1 oligomerization equilibria in solution and promotes higher-order Drp1 oligomerization, results wholly consistent with previous observations^19,27,28^.

We used negative-stain electron microscopy (EM) to determine whether GFP-tagging affected either the geometry and/or the efficacy of GTP-dependent Drp1 helical self-assembly in solution. In the presence of GMP-PCP, Drp1-*wt* constituted an equitable mixture of rings (shorter oligomers) and helices (longer oligomers) (Fig. 3C). By contrast, mEGFP-Drp1 almost exclusively constituted helices (Fig. 3D), which were of a slightly larger diameter than that of Drp1-*wt* (Fig. 3E), a difference attributable to the extra mEGFP moiety. Importantly, mEGFP-Drp1 helices were conspicuously longer than that of Drp1-*wt* (Fig. 3F). Together, these data indicated that GFP-tagged Drp1 not only adopts a larger oligomeric state relative to Drp1-*wt* in the absence of nucleotide in solution but also has a greater propensity for higher-order helical polymerization upon GTP binding, in vitro.

### GFP-tagged Drp1 exists primarily as a tetramer in the cytosol

Raster-image correlation spectroscopy (RICS) is an imaging-based analytical technique that can accurately measure D as well as determine the concentrations of fluorescently labeled biomolecules with high spatiotemporal resolution in living cells^43,44^. By contrast to single-point FCS which strictly utilizes a temporal correlation of fluorescence intensity fluctuations, RICS also employs a spatial autocorrelation based on the stacking of time-stamped confocal images obtained by the laser scanning confocal microscope using the ‘raster scanning’ mode^45–47^. RICS is better suited to measure D in living cells as it better defines the probability of finding a fluorescently labeled particle at different times and locations^43^.

**Figure 4:**
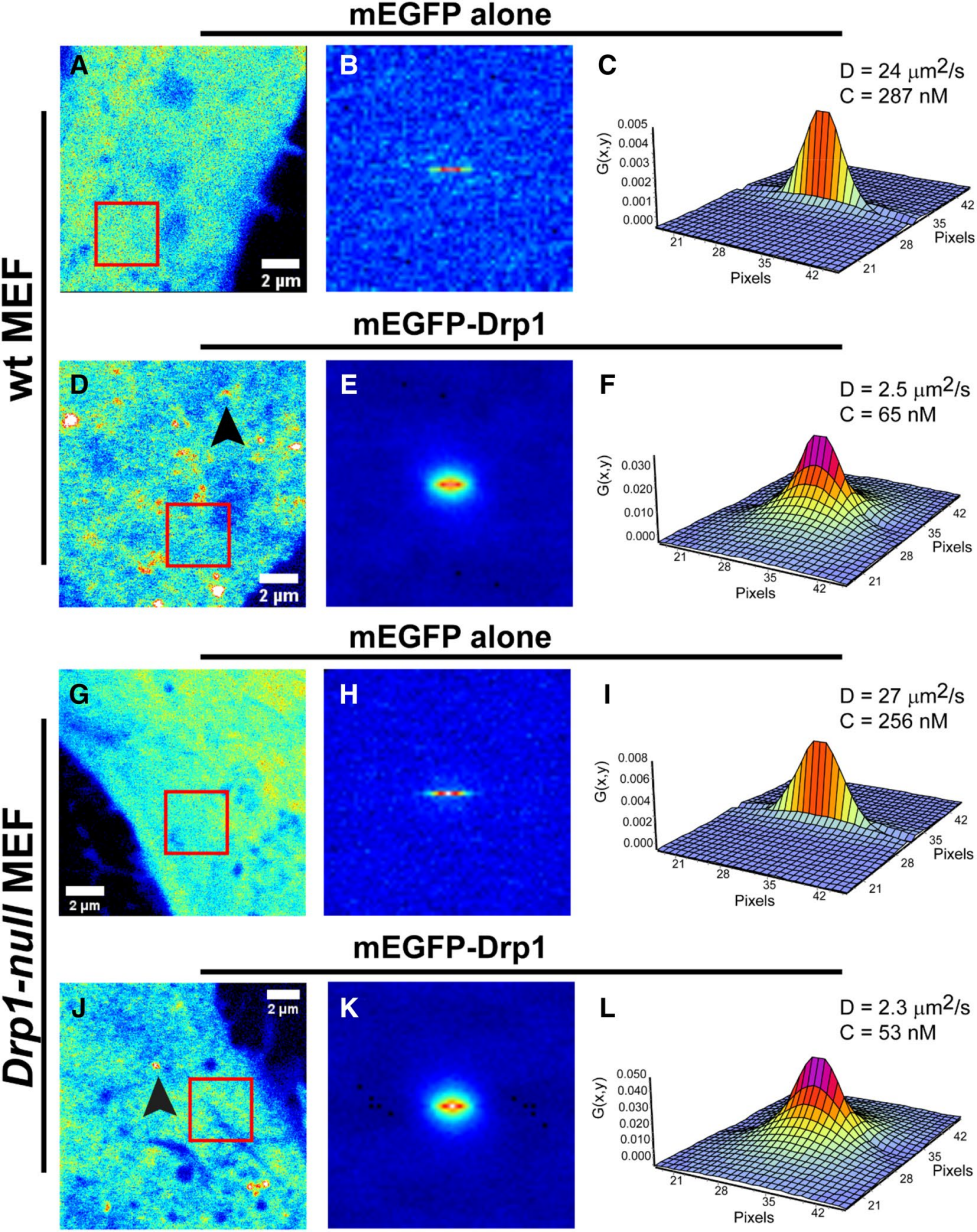
RICS analysis reveals a higher-order oligomeric state for mEGFP-Drp1 in vivo. D and concentration of cytosolic mEGFP-Drp1 expressed in representative *wt* and *Drp1-null* MEFs determined using RICS. All RICS data statistics are shown in Table 2. For reference, mEGFP alone was expressed and analyzed in parallel experiments. The *left* panels (**A**,**D**,**G**,**J**) show the average background-corrected fluorescence intensity recovered from a stack of 100 frames (12.8 × 12.8 μm) collected in each experiment. Arrowheads indicate the localization of bright puncta in mEGFP-Drp1-expressing cells. The red box (3.2 × 3.2 μm) indicates the region where a localized RICS analysis was performed, typically at the cell periphery. The *center* panels (**B**,**E**,**H**,**K**) display the image of the spatial correlation function obtained from the localized RICS analysis (red box). The *right* panels (**C**,**F**,**I**,**L**) show the fits of the spatial correlation function that retrieve the best-fit D and the particle concentration of the fluorescent molecules (see “Methods” for additional details). D of mEGFP varies in the range, D_mEGFP_ = 20–30 μm^2^/s in different regions of the cell periphery, whereas that of mEGFP-Drp1 varies in the range, D_mEGFP-Drp1_ = 1.0–3.0 μm^2^/s. All images in this figure were obtained using the SimFCS 4 software available at https://www.lfd.uci.edu/globals/.

We used RICS to measure D of mEGFP-Drp1 in comparison to mEGFP alone in transfected *wt* and *Drp1-null* MEFs^21,48^. As expected, mEGFP alone was distributed throughout the cell, including the nucleus (Supporting Fig. S2A,B). In contrast, mEGFP-Drp1 (~ 111 kDa monomeric size) was restricted to the cytoplasm and was observed as both diffuse species and largely immobile puncta associated with mitochondria (Supporting Fig. S2C,D). Localized RICS analysis at the less-crowded and better-resolved cell periphery region to avoid interference from bright, immobile puncta associated with clustered perinuclear mitochondria, which introduces artifacts to the fluctuation data, revealed marked differences in diffusion between mEGFP and cytosolic mEGFP-Drp1 as assessed by the inspection of their spatial autocorrelation plots (spatial autocorrelation function or ACF) (Fig. 4). For mEGFP alone, the spatial ACF was elongated in the horizontal direction owing to its relatively fast diffusion (Fig. 4B,H). In contrast, the spatial ACF for mEGFP-Drp1 showed broadening in both the horizontal and vertical directions indicating a much slower diffusion (Fig. 4E,K). Fits of the spatial ACF for mEGFP alone (Fig. 4C,I) estimated D in the range 20–30 μm^2^/s with particle number concentrations in the range of 200–400 nM (Table 2). These values for mEGFP were in good agreement with previous reports^43,45,47^. For mEGFP-Drp1, fits of the spatial AFC (Fig. 4F,L) yielded D in the range 1.0 to 3.0 μm^2^/s. These data indicated a significantly larger size for cytosolic mEGFP-Drp1, which on average had a ~ tenfold lower D than mEGFP, and particle number concentrations in the 20–150 nM range (Table 2). The remarkable similarity in these numbers between mEGFP-Drp1 obtained here and similarly sized family member dynamin 2 (Dnm2-mEGFP) obtained previously^49^, suggested that mEGFP-Drp1, like Dnm2-mEGFP, is predominantly a tetramer in the cytosol. However, diffusion rates seldom correlate with the molecular aggregation state largely due to the convoluted dependency of the diffusion coefficient on molecular hydrodynamic volume, and also because the anisotropic environment of the cytoplasm differentially influences protein diffusion in unpredictable ways^43,45,47^.

**Table 2:** Diffusion coefficients (D) and particle number concentrations (C) for cytosolic mEGFP alone and mEGFP-Drp1 overexpressed in live cells and measured using RICS.

	D, μm^2^/s	C, nM	n
mEGFP alone (HeLa)	22.4 ± 3.29	345 ± 207	11
mEGFP-Drp1 (HeLa)	1.54 ± 0.72	115 ± 95.4	13
mEGFP alone (*wt* MEF)	26.2 ± 1.49	331 ± 57.9	10
mEGFP-Drp1 (*wt* MEF)	2.18 ± 0.55	58.5 ± 33.6	12
mEGFP alone (Drp1-*null* MEF)	23.9 ± 3.57	375 ± 189	13
mEGFP-Drp1 (Drp1-*null* MEF)	2.45 ± 0.65	25.9 ± 7.77	11

*n* number of cells measured (one RICS measurement per cell).

Values reported are mean ± S.D.

N&B analysis, a diffusion-independent complementary approach^46,47,50^ based on relative molecular brightness, was therefore used to unambiguously determine the oligomerization state(s) of mEGFP-Drp1 in the cytosol utilizing the same image stack obtained for RICS (see Methods for a detailed description). By comparing the brightness of each pixel of mEGFP-Drp1 to that of mEGFP alone (a known monodisperse monomer), the oligomeric state of mEGFP-Drp1 can be determined. An identical approach was previously used for Dnm2-mEGFP^49^.

**Figure 5:**
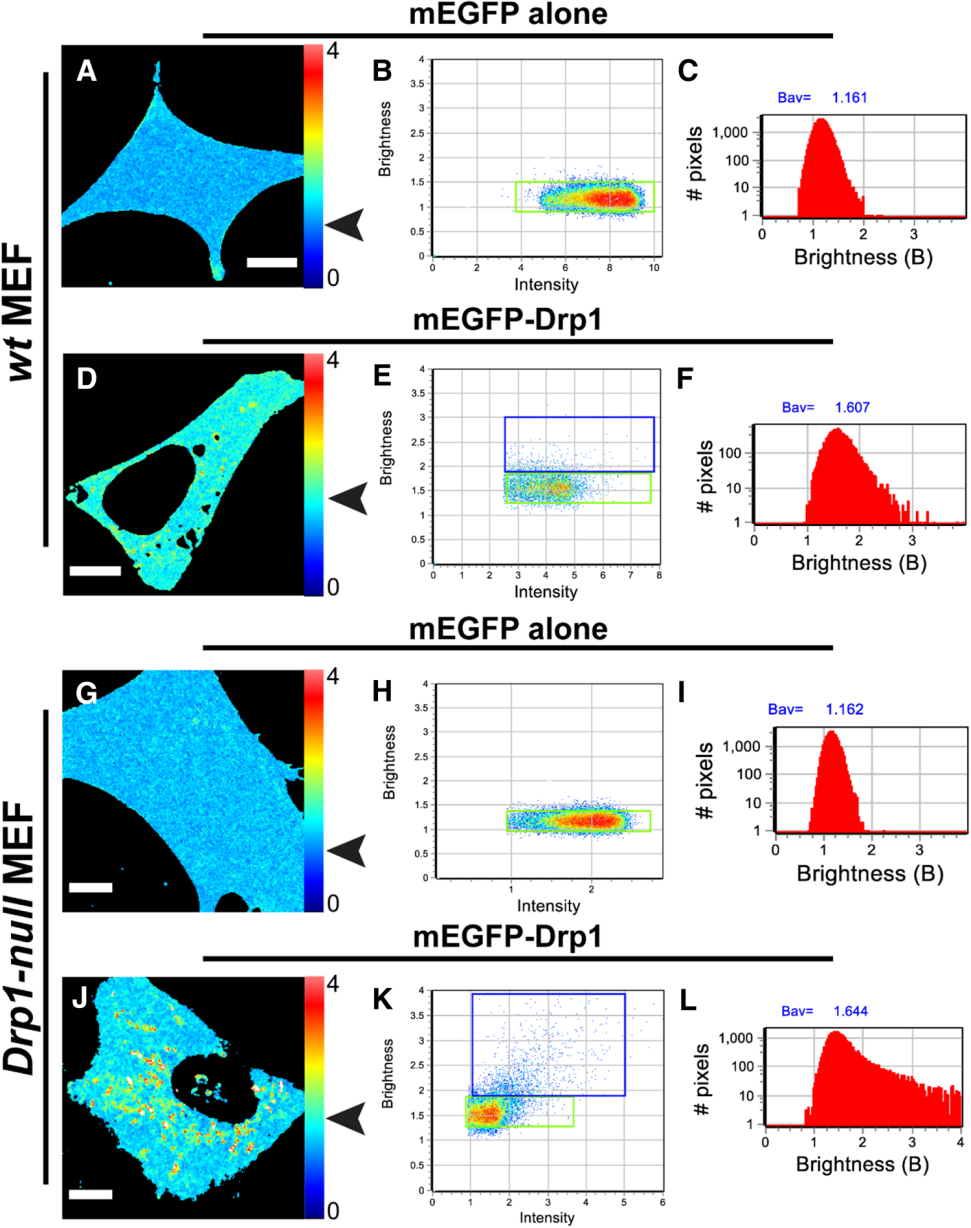
N&B analysis reveals that cytosolic mEGFP-Drp1 lacks oligomerization dynamics. Molecular brightness of mEGFP-Drp1 expressed in *wt* and *Drp1-null* MEFs determined using N&B analysis. *Left* panels (**A**,**D**,**G**,**J**) show the distribution of background-subtracted B-values across the cell body. The pixel color corresponds to B-values scaled from 0 to 4. 100 frames were collected (~ 50 × 50 μm) for each experiment. *Center* panels (**B**,**E**,**H**,**K**) show the collection of pixels of the B-map (left image) plotted according to their B-values versus the fluorescence intensity. For mEGFP, the green box in (**B**) and (**H**) selected the pixels with average B ~ 1.16 in both *wt* and *Drp1-null* MEFs (indicated by arrowheads in panels **A** and **G**, respectively). The histogram of B-values of the pixels corresponding to cytosolic mEGFP (**C**,**I)** shows a Gaussian distribution in both cell types. For mEGFP-Drp1, the green box in (**E**) and (**K**) selected the pixels with average B = 1.59 in *wt* MEFs (indicated by arrowhead in **D**) and average B = 1.61 in *Drp1-null* MEFs (indicated by arrowhead in **J**). The histograms of B-values for mEGFP-Drp1 (**F**,**L**) show a broadening towards higher values with a maximum centered at B = 1.65 ± 0.25 for *wt* MEFs and B = 1.64 ± 0.32 for *Drp1-null* MEFs when fitted to a Gaussian distribution). The blue boxes selected pixels with average B-values of 2.45 and 2.59, respectively, for *wt* and *Drp1-null* MEFs. Scale bar, 10 μm. All images in this figure were obtained using the SimFCS 4 software available at https://www.lfd.uci.edu/globals/.

**Figure 6:**
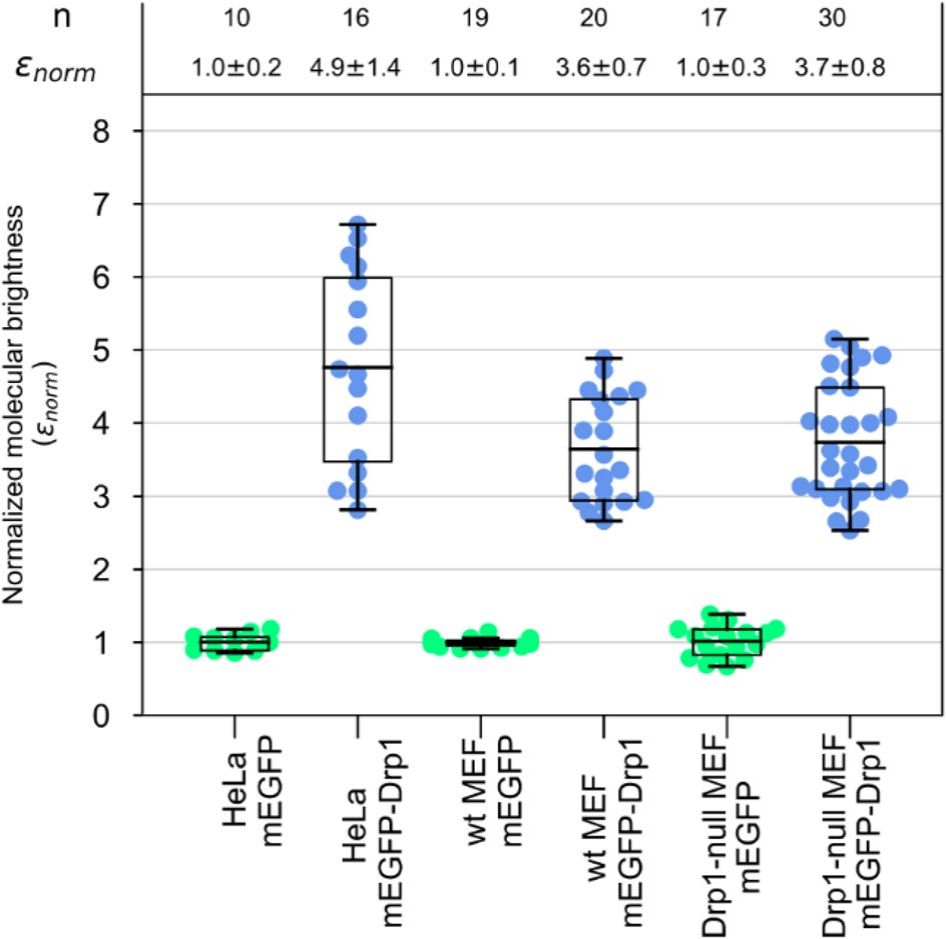
Cytosolic mEGFP-Drp1 is primarily a tetramer. The normalized true molecular brightness (ε_*norm*_) of mEGFP and mEGFP-Drp1 determined in three mammalian cell lines compared using box plots. The boxes enclose 50% of the data points obtained (solid circles), and the whiskers show the maximum stretch of the data. Each data point corresponds to one measured cell. The horizontal lines in each box represent the mean values of the normalized data. The molecular brightness values are normalized to that of mEGFP in each cell line. Therefore, the ε_norm_ is equal to the number of subunits present. The top panel reports the number of measurements (n) performed for each cell line, and the ε_norm_ average ± S.D. for each group. Box and whisker plots were prepared using matplotlib v3.2.2.

For our N&B analysis, we selected *wt* and *Drp1-null* MEFs expressing relatively low concentrations of mEGFP-Drp1 to avoid high expression artifacts such as saturating bright puncta resulting from cytosolic protein aggregation. A comparison of the distribution of brightness (B)-values projected on the images of representative cells revealed the differences between the brightness of mEGFP alone and mEGFP-Drp1 (Fig. 5). The distribution of B-values across the cell body for mEGFP-Drp1 was however non-homogeneous (Fig. 5D,J). Some areas showed signal saturation, or simply a lack of fluctuations in fluorescence intensity (represented by white or black pixels). Puncta associated with mitochondria were relatively stable during the acquisition of the image stacks and were duly eliminated from further analysis. The pixels corresponding to cytosolic mEGFP-Drp1 (green boxes; Fig. 5E,K) showed a relatively small spreading of B-values, but accounted for more than 90% of the pixels. As expected, the histogram of B-values for mEGFP-Drp1 was strikingly different from that of mEGFP alone (Fig. 5F,L). In both *wt* and *Drp1-null* MEFs, the histograms showed a marked broadening towards higher B-values for mEGFP-Drp1 indicating a larger oligomerization state compared to mEGFP alone. Quantitative estimation of the brightness of mEGFP-Drp1 made by fitting the B-value histograms to a Gaussian distribution, which was achieved by centering the distribution at the maximum values, resulted in B-values of 1.65 ± 0.25 for *wt* MEFs and 1.64 ± 0.32 for *Drp1-null* MEFs. The molecular brightness value (ε) derived for mEGFP-Drp1 by normalizing its B-values to that of mEGFP alone (Fig. 6) was ~ 4 indicating that mEGFP-Drp1 is almost exclusively a tetramer in the cytosol, and lacks oligomerization dynamics, much in keeping with the biochemical and biophysical data obtained above.

### Persistent GFP-tagged Drp1 self-assembly divides mitochondria in situ

We next investigated whether the altered quaternary structure and oligomerization propensity of GFP-tagged Drp1, bereft of robust native Drp1-like GTPase activity and dynamics, somehow affected mitochondrial fission in situ. Although mEGFP-Drp1 and Drp1-mEGFP have both been previously demonstrated to participate in mitochondrial fission^51–53^, their assembly-disassembly dynamics on mitochondria and efficacy in mitochondrial fission, especially in the absence of co-mixed native Drp1, have not been thoroughly investigated or quantified. To this end, we expressed mEGFP-Drp1 in *wt* and *Drp1-null* MEFs.

**Figure 7:**
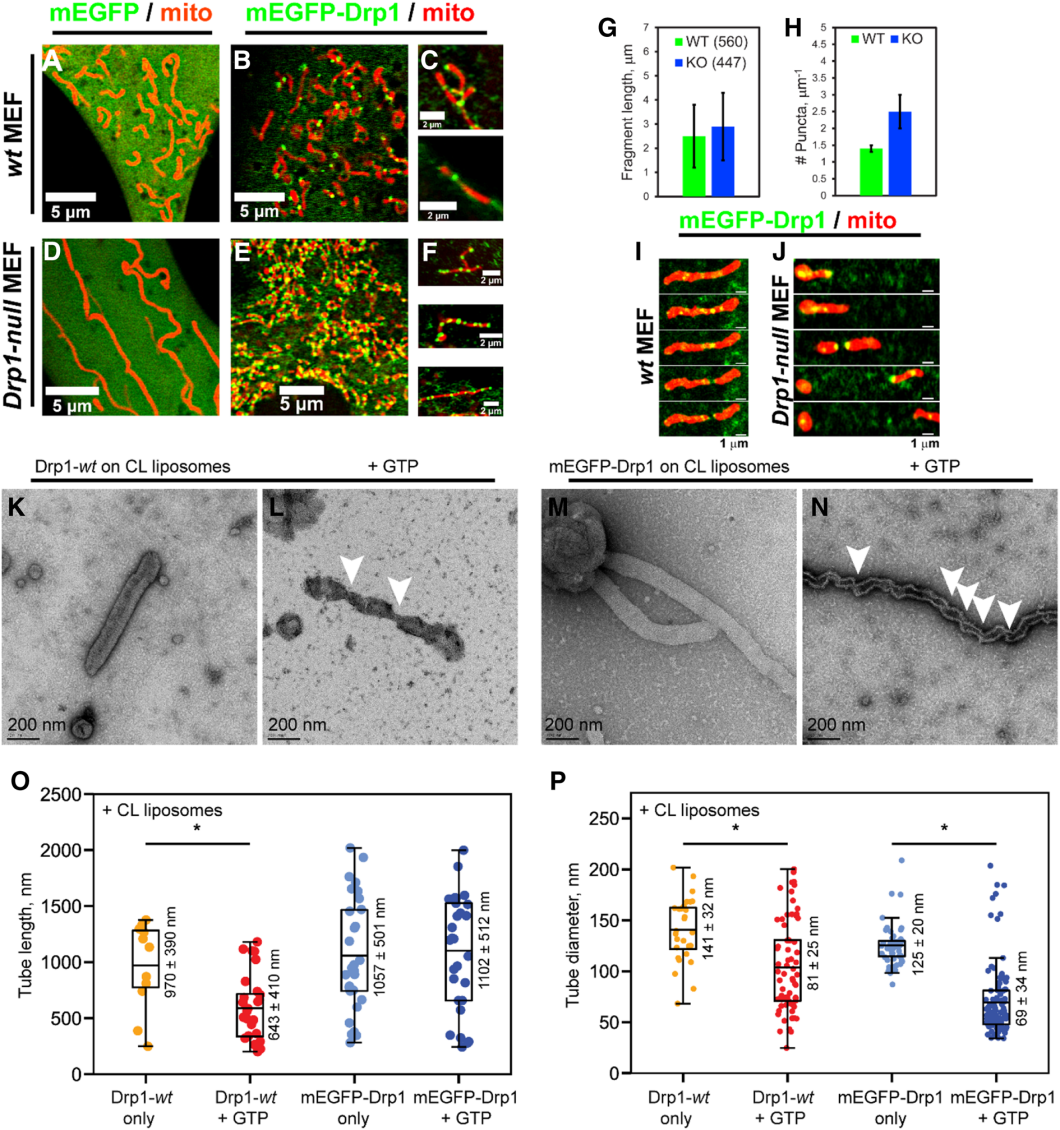
Persistent mEGFP-Drp1 self-assembly favors mitochondrial fission. Confocal fluorescence images of *wt* (**A**–**C**) and *Drp1-null* MEFs (**D**–**F**) expressing mEGFP alone or mEGFP-Drp1 (green). Mitochondria were visualized using mCherry-Mito-7 (red). Compared to *wt* MEFs, Drp1-null MEFs displayed a greater number of mEGFP-Drp1 puncta per unit length of mitochondria (**C**,**F**). (**G**) Mitochondrial fragment lengths in mEGFP-Drp1-expressing *wt* and *Drp1-null* (knockout or KO) MEFs. Numbers in parenthesis indicate the number of mitochondrial fragments counted from at least five cells. (**H**) Frequency of mEGFP-Drp1 puncta per μm mitochondrial fragment length in *wt* and *Drp1-null* (KO) MEFs. (**I,J**) Time-lapse images of mitochondrial fission mediated by mEGFP-Drp1 in *wt* (panel **I**) and *Drp1-null* MEFs (**J**). (**K**–**N**) Representative negative-stain EM images of CL-containing liposomes tubulated by Drp1-*wt* and mEGFP-Drp1 before (**K**,**M**) and after addition of GTP (**L**,**N**). Arrowheads in panels (**L**) and (**N**) point to regions of membrane tube superconstriction approaching membrane hemi-fission. Scale bar, 200 nm. (**O**,**P**) Boxplots of membrane tube lengths (**O**) and diameters (**P**) for Drp1-*wt* and mEGFP-Drp1 in the absence and presence of GTP. Statistically significant differences between groups are indicated by a star. Mean ± S.D. values are indicated next to each box. For membrane tube lengths in panel **O**, Drp1-*wt* only n = 11, Drp1-*wt* + GTP n = 29, and p = 0.0281; mEGFP-Drp1 only n = 30, mEGFP-Drp1 + GTP n = 29, and differences are not significant. For membrane tube diameters in panel **P**, Drp1-wt only n = 28, Drp1-*wt* + GTP n = 69, and p < 0.0001; mEGFP-Drp1 only n = 56, mEGFP-Drp1 + GTP n = 116, and p < 0.0001. Fluorescence images were prepared using the software platform Fiji^35^. Bar and box and whisker plots were prepared using matplotlib v3.2.2.

As earlier, the overexpression of mEGFP-Drp1, but not of mEGFP alone, robustly restored mitochondrial fragmentation in *Drp1-null* MEFs, which otherwise displayed elongated and hyperfused mitochondria^21^ (Fig. 7A–F). A comparison of resultant mitochondrial fragment lengths between mEGFP-Drp1-overexpressing *wt* and *Drp1-null* MEFs revealed that their mitochondrial dimensions were vastly similar (Fig. 7G). Remarkably, however, a 50% greater incidence of mEGFP-Drp1 puncta was found over mitochondrial fragments in *Drp1-null* MEFs compared to *wt* MEFs (Fig. 7H). These data indicated that mEGFP-Drp1, in the absence of native Drp1 and a robust GTP hydrolysis rate, forms disassembly-refractory oligomers over mitochondria. Despite this, however, seemingly normal mitochondrial fission was observed in mEGFP-Drp1-overexpressing *Drp1-null* MEFs relative to *wt* MEFs (Fig. 7I,J).

EM results obtained with mEGFP-Drp1 on CL-containing liposomes in vitro validated our supposition that mEGFP-Drp1 oligomers were indeed refractory to GTP hydrolysis-driven disassembly. We anecdotally noted that a greater incidence of, and longer, membrane tubes resulted from the helical self-assembly of mEGFP-Drp1 on CL-containing liposomes relative to Drp1-*wt*. In addition, mEGFP-Drp1-decorated membrane tubes also remained persistently longer upon GTP addition in comparison to Drp1-*wt* (Fig. 7K–N). Quantification of tube lengths indicated that whereas membrane-bound Drp1-*wt* oligomers underwent some degree of GTP hydrolysis-dependent disassembly leading to tube shortening (Fig. 7O), those of mEGFP-Drp1, on average, did not. Furthermore, in the presence of GTP, mEGFP-Drp1 oligomers constricted the underlying membrane tubes to a smaller diameter more frequently than Drp1-*wt* (Fig. 7P). These data explained the greater incidence of narrow, superconstricted membrane tube regions approaching hemi-fission observed for mEGFP-Drp1 relative to Drp1-*wt* (Fig. 7N,O).

Taking into account the suppressed GTP sensitivity and GTPase activity of mEGFP-Drp1, these data together indicate that persistent Drp1 self-assembly on membranes is sufficient for mitochondrial fission. Alternatively, although not mutually exclusively, these data also indicate that a significant proportion of mEGFP-Drp1 oligomers formed over mitochondria in *Drp1-null* MEFs are likely kinetically trapped and are hence non-productive for fission, thus explaining the similar extents of mitochondrial fission observed between the two different cell types. Regardless of these differences and mechanisms, we conclude that GFP-tagged Drp1 is not a true surrogate of native Drp1, which may explain the various confounding results that inform current models of Drp1-catalyzed mitochondrial fission.

## Discussion

Here we sought to address longstanding controversies in the field surrounding Drp1′s oligomerization state(s) in solution, selective recruitment to mitochondria, and the role of GTP-driven assembly-disassembly dynamics in mitochondrial fission. Using quantitative imaging techniques and supporting biophysical measurements, we report on the serendipitous discovery that many of the above conflicts in the published literature stem from the use of disparately labeled Drp1 fluorescent constructs, including the widely disseminated but flawed GFP-tagged Drp1. We implore that any structural or mechanistic information derived from the use of GFP-tagged Drp1, either previously^23,24,28,30^ or in studies henceforth, be interpreted with an abundance of caution.

It has been long recognized that only a small subset of all Drp1 foci formed over mitochondria progress to a productive fission event^24,51,54,55^. While this suggests that Drp1 traverses various regulatory checkpoints that precisely control the timing and efficiency of mitochondrial fission, our data indicate that this is likely an erroneous estimate emanating from the use of GFP-tagged Drp1, which we now demonstrate constitutes GTP-resistant, supramolecular assemblies, both in solution and on membranes. We suggest that the subdued GTP-driven assembly-disassembly dynamics of GFP-tagged Drp1 manifests as the great majority of non-productive Drp1 puncta in vivo.

More recently, cytosolic tetramers and higher-order oligomers of Drp1, but not dimers, were purported to be selectively recruited by Mff to mitochondria in vivo^23,24,28^, a conclusion drawn in apparent contrast to previous in vitro studies that demonstrated the contrary i.e. a selective binding of Drp1 dimers, but not of higher-order oligomers, by Mff^22,29^. Again, we reason that this is largely owing to the use of GFP-tagged Drp1 in the cellular studies, which in contrast to native Drp1 does not equilibrate to minimal Drp1 dimers either in vitro or in vivo.

It likewise did not escape our attention that native or overexpressed Myc-tagged Drp1 puncta are found relatively less abundantly over mitochondria upon immunostaining^21,51,56^, in comparison to GFP-tagged Drp1, which shows an overabundance of mitochondria-associated foci^51,54^. Moreover, GFP-tagged Drp1 puncta seemingly persist over mitochondrial division sites at the time of fission and undergo splitting to partition with both of the newly formed mitochondrial poles post-division^32,57^. We sound caution that this is likely an aberrant result caused by the enhanced stability of GFP-tagged Drp1 oligomers over mitochondria, likely as a consequence of the drastically suppressed GTPase activity and impaired oligomer disassembly. Interestingly, GFP variant rsKame-tagged Drp1 does not reproduce such behavior and partitions with only one of the mitochondrial poles in vivo^58^. This is consistent with the notion that the intrinsic properties of the GFP moiety can influence target protein aggregation^7^, and in the case of GFP-tagged Drp1, also its responsiveness to GTP. Nevertheless, the capacity of dysregulated GFP-tagged Drp1 to mediate membrane fission both in vitro and in vivo suggests that GTP-insensitive Drp1 higher-order self-assembly, in the absence of robust assembly-disassembly dynamics, can be a driver of membrane fission. The physiological relevance of this non-native fission mechanism however remains highly debatable. Indeed, the artificial crowding of GFP alone on membranes produces similar artifacts, including membrane tubulation and fission in vitro^59^.

On a technical note, the D of mEGFP-Drp1 was recently measured in HeLa cells using single-point FCS^19^. The reported values in the range 6–9 μm^2^/s are significantly different (signifying faster diffusion) from those obtained in our study. We reason that while single-point FCS may be sufficient to determine the D of isotropically diffusing, globular particles (e.g. mEGFP alone) in a homogeneous medium, it is inadequate and likely misleading for Drp1, an elongated molecule that diffuses in the anisotropic environment of the cytoplasm. The Stokes–Einstein equation that relates D to molecular mass for strictly globular particles, therefore, does not hold true for Drp1. In the case of Drp1, particle mobility depends on time and on position for which RICS, as employed here, and not single-point FCS as used previously, is better suited for a precise determination of D in vivo.

Our investigation also discards any cell type-specific differences in Drp1 oligomerization dynamics and diffusion behavior. In HeLa cells where GFP-tagged Drp1 expression was considerably higher than in MEFs, GFP-tagged Drp1 puncta not only localized on mitochondria but also appeared in the cytoplasm with no apparent association with mitochondria (Fig. 1B). For our RICS and N&B analyses, we picked HeLa cells comparable in Drp1 expression to the MEFs (Supporting Figs. S3,S4). We found that for both mEGFP and GFP-tagged Drp1, the recovered D and ε from the two different cell types are remarkably similar to those observed between *wt* and *Drp1-null* MEFs. We conclude that the diffusional properties of GFP-tagged Drp1 are not cell line-dependent.

In summary, the utility of GFP- and dye-labeled proteins as surrogates to discern native protein behavior and function either in vitro or in vivo requires a thorough investigation of their biochemical, biophysical, and cellular properties in direct comparison with their unlabeled, native counterparts. The lessons learned from this study caution against the presumption that GFP-tagging rarely interferes with native protein behavior and/or function. Nevertheless, our studies with GFP-tagged Drp1 reveal that persistent but dysregulated Drp1 self-assembly (crowding) over membranes can act as a potent driver of mitochondrial fission, and perhaps, of all self-assembled protein-mediated membrane fission, as demonstrated for nonenzymatic BAR domain-containing proteins recently^60^.

## Materials and methods

### Protein expression, purification and fluorescence labeling

Drp1-mEGFP and mEGFP-Drp1 constructs for mammalian cell expression were made by sub-cloning DNA encoding human Drp1 isoform 3 into pEGFP-N1 and pEGFP-C1 (Clontech), respectively. The EGFP-to-mEGFP converting mutation A206K^61^ (also denoted as A207K in the literature) was introduced by site-directed mutagenesis. Corresponding constructs for *E. coli* expression were made by sub-cloning the fusion constructs from the above mammalian expression plasmids into pET28a (Novagen). pET28a appends an N-terminal 6XHis-tag for protein purification. All constructs were verified by automated DNA sequencing. N-terminal 6XHis-tagged Drp1-*wt*, mEGFP, mEGFP-Drp1, and Drp1-mEGFP were expressed recombinantly in *E. coli* and purified to apparent homogeneity as previously described^21^. Proteins were additionally cleaned up by gel-filtration chromatography through an ENrich SEC 650 10 × 300 mm column (Bio-Rad). Protein concentrations were determined by absorbance at 280 nm. Drp1 was fluorescently labeled with thiol-reactive BODIPY-FL iodoacetamide or AF488 maleimide (ThermoFisher Scientific) as previously described^62^. Among the 9 total Cys in Drp1, the primary targets of thiol modification are surface residues C505 and C607 located in the disordered VD^31^. The stoichiometry of labeling was determined by absorbance using ε_502_ of 76,000 M^−1^ cm^−1^ and ε_495_ of 72,000 M^−1^ cm^−1^ for BODIPY-FL and AF488, respectively. Labeling efficiency was ~ 2 mol dye/mol protein in both cases. Protein stocks were aliquoted and stored at − 80 °C in storage buffer (20 mM HEPES pH 7.5, 150 mM KCl, and 1 mM DTT) containing 10% glycerol.

### GTPase assay

GTP turnover (k_cat_) at 37 °C in the absence (basal; 1-h incubation after GTP addition) and presence of CL-containing liposomes (CL-stimulated activity; 12-min incubation after GTP addition) was measured using the malachite green-based colorimetric assay as previously described^21^. Data represent the average of three independent experiments ± S.D.

### SEC-MALS

SEC elution profiles, differential refractive indices, and molar mass distributions were obtained as previously described^21^. Proteins were fractionated on a Superose 6 10/300 GL column (GE Healthcare, Piscataway, NJ) in buffer containing 20 mM HEPES pH 7.5, 150 mM KCl, and 1 mM DTT and analyzed using in-line miniDAWN Treos MALS and Optilab rEX differential refractive index detectors (Wyatt Technologies, Santa Barbara, CA). Data analysis was performed using the ASTRA 6.1 software package (Wyatt Technologies). The loading protein concentrations were varied as indicated and injected in a volume of 500 μL.

### Negative-stain EM

Drp1-*wt* and mEGFP-Drp1 (2 μM protein final) incubated either with GMP-PCP (1 mM final) or with CL-containing liposomes (50 μM total lipids final) before and after the subsequent addition of GTP (1 mM final), were stained and imaged using a Tecnai Spirit BioTwin transmission electron microscope (FEI, Eindhoven, Netherlands) operated at 100 keV as previously described^17,21^. Images were acquired using a Gatan US4000 UHS charge-coupled device (CCD) camera (4 k × 4 k) (Gatan, Warrensdale, PA).

### Statistical analyses of Drp1 helical and membrane tube diameters and lengths

Box and whisker plots were built using matplotlib version 3.2.2 available at https://matplotlib.org/3.2.2/api/_as_gen/matplotlib.pyplot.boxplot.html. The box extends from the lower to the upper quartile values of the data (25^th^-75^th^), and the horizontal line is plotted at the median value of the data set. The whiskers show the full range of the data extending from the minimum to the maximum value of the data set. Comparisons of the distributions of lengths and diameters were made using the unpaired t-student test.

### Cell biology

*wt* and *Drp1-null* mouse embryonic fibroblast (MEF) cells^48^ and human cervical adenocarcinoma (HeLa S3) cells were grown in DMEM supplemented with 10% heat-inactivated fetal bovine serum and 1% penicillin/streptomycin at 37 °C in 5% CO_2_ atmosphere. Before imaging, the cells were plated at 50–60% confluency in glass-bottom microscopy dishes (35 mm, N° 1.5 glass, 14 mm well-diameter, MatTek, Ashland, MA) pre-coated with fibronectin/gelatin solution (10 μg/mL/20 μg/mL). Cells were transfected with 1–2 μg of plasmid DNA encoding either mEGFP alone or the mEGFP Drp1 fusion constructs together with the mCherry-Mito-7 plasmid (gift from Michael Davidson, Addgene plasmid #55102) using the Lipofectamine LTX kit (Invitrogen) according to the manufacturer’s protocol. Cells were incubated for 24–48 h post-transfection. One hour before imaging, the culture medium was exchanged with Fluorobrite DMEM media (Gibco) supplemented with 1X GlutaMAX and 10 mM HEPES, pH 7.2 (Gibco).

### Confocal microscopy

Confocal images and fluorescence fluctuation data were acquired with an Olympus FluoView FV1000 laser scanning microscope (Olympus Corporation, Center Valley, PA) and a PlanApo N 60X 1.42 NA oil-immersion objective (Olympus) using 488 nm excitation for mEGFP, BODIPY-FL, and Alexa-488, and 543 nm excitation for mCherry. The primary dichroic filter for excitation was 405/488/559/635, and for emission, the band pass filters used were 505–525 nm and 560–660 nm for the green and red channels, respectively. The pinhole was set at 95–105 μm. Lasers were set at 1% output (using the software control slider) unless otherwise stated. Detectors were set to the pseudo-photon counting mode. High-resolution images of cells were acquired at 1,024 × 1,024 pixels using Kallman averaging every two lines. Cell culture dishes were mounted on a heated stage set at 37 °C and imaged for < 4 h.

### FCS

FCS experiments were performed at 25 °C. Fluorescently labeled protein samples were pre-equilibrated in assay buffer (20 mM HEPES, pH 7.5, 150 mM KCl, 1 mM DTT ) at their desired final concentration (0.5 μM) upon dilution from protein stocks. Additives in the storage buffer were removed by buffer exchange using gel filtration microcolumns (Zeba, 7 K MWCO, Thermofisher Scientific). In pertinent samples, GMP-PCP and MgCl_2_ were added to 1 mM final each  and equilibrated for 30 min before data acquisition. For data acquisition, the pixel time was set to 10 μs. Data were collected using the crosshair ROI selector placed at the center of the frame and 32,766 data points were acquired in the “Freerun” mode (89 s acquisition time) with the digital zoom set to 1. The point-spread function (PSF) was calibrated using a solution of 100 nM AF488 with a known D of 435 μm^2^/s^63^ (Supporting Fig. S5). The resulting radial waist (ω_r_) varied in the range 0.21–0.28 μm on a day-to-day basis. The data files were saved using the FluoView software (3.1.3.3), and exported as 16-bit “tif” format files. Subsequently, the tif files were loaded onto the SimFCS software (Laboratory for Fluorescence Dynamics, www.lfd.uci.edu). Raw data were analyzed with the large vector correlation module, and the autocorrelation data were fit with the built-in tools.

For a 3D Gaussian excitation volume, the autocorrelation function G (τ) that describes the diffusion of a single particle is:

1 $$G_{FCS} \left( \tau \right) = \frac{\gamma }{N}\left( {1 + \frac{\tau }{{\tau_{D} }}} \right)^{ - 1} \cdot \left( {1 + S^{2} \cdot \frac{\tau }{{\tau_{D} }}} \right)^{{ - \frac{1}{2}}}$$

where τ is the time correlation shift, γ = 0.3536 is the geometric factor accounting for the shape of the excitation volume with one-photon excitation, N is the number of fluorescent particles in the excitation volume, τ_D_ is the diffusion time that is related to the diffusion coefficient, D, by τ_D_ = ω_r_^2^/4D. The structural factor S = ω_r_/ω_z_ relates the axial (ω_z_) and radial (ω_r_) waists of the PSF. The autocorrelation function extrapolated to time zero is a fitting parameter that takes the value G(0) = γ/N, the amplitude of the autocorrelation function. The volume of the PSF is calculated using^64^:

2 $$V_{3DG} = \omega_{r}^{2} \cdot \omega_{z} \cdot \left( {\frac{\pi }{2}} \right)^{\frac{3}{2}} = \left( \frac{1}{2} \right)^{\frac{3}{2}} \cdot V_{eff}$$

The effective observation volume can be calculated from the best-fit value for G(0), Avogadro’s number, N_A_, and the concentration of the fluorophore, C, as:

3 $$V_{eff} = \frac{1}{{G\left( 0 \right) \cdot N_{A} \cdot C}}$$

The diffusion coefficient of a molecule in an aqueous environment is related to its hydrodynamic radius, r_H_, by the Stokes–Einstein equation:

4 $$D = \frac{kT}{{6\pi \eta r_{H} }}$$

where *k* is the Boltzmann constant, T is the absolute temperature, η is the viscosity of the solvent^38^. D of mEGFP and BODIPY-FL in solution under our experimental conditions were 98 μm^2^/s (Supporting Fig. S6) and 476 μm^2^/s (Fig. 2D), respectively.

### RICS

RICS stacks were collected using 12.5 μs/pixel, 256 × 256 pixel frame size, and 0.05 μm pixel size (digital zoom set to 16.4). A stack of 100 frames was collected in each experiment with a total acquisition time of 115 s. Calibration of the PSF was performed using a solution of 100 nM AF488 at 25 °C with 8 μs pixel time (Supporting Fig. S7). Fits of the radial waist (ω_r_) varied between 0.23–0.30 μm on a day-to-day basis. All live cell measurements were made at 37 °C. Data analysis was performed with the SimFCS software (Laboratory for Fluorescence Dynamics, www.lfd.uci.edu). Corrections for cell and organelle motions were introduced by applying a background subtraction with a moving average filter of 7–10 frames.

The basic equation that describes the spatial autocorrelation due to the diffusion of fluorescent molecules takes the form^43^:

5 $$G_{RICS} \left( {\xi ,\psi } \right) = \frac{\gamma }{N}\left( {1 + \frac{{4D\left( {\tau_{p} \xi + \tau_{l} \psi } \right)}}{{\omega_{r}^{2} }}} \right)^{ - 1} \cdot \left( {1 + \frac{{4D\left( {\tau_{p} \xi + \tau_{l} \psi } \right)}}{{\omega_{z}^{2} }}} \right)^{{ - \frac{1}{2}}}$$

ξ and ψ are the horizontal and vertical spatial correlation pixel shifts, γ = 0.3536 is the geometric factor for one-photon excitation, N is the number of molecules, τ_p_ is the pixel dwell time, τ_l_ is the time between lines.

### N&B analysis

N&B stacks were acquired using a frame size of 256 × 256 pixels, 12.5 μs pixel time, and 0.05–0.2 μm pixel size. 100 images were acquired for each experiment in about 2 min. The laser power was set at 1% for all acquisitions. The N&B analysis utilizes the intensity fluctuations on each pixel of an image produced by the diffusion of fluorescent molecules allowing the distinction of pixels with many dim molecules from pixels with few bright molecules. N&B calculates the apparent brightness (B) and the apparent number of particles (N) using moment analysis of the fluorescence intensity distribution on each pixel of the image. Data analysis was performed with SimFCS software (Laboratory for Fluorescence Dynamics, www.lfd.uci.edu). For the Olympus FluoView FV1000 analog detector operating in the pseudo-photon counting mode, these parameters are calculated from the average and the variance of the recorded intensity distribution at each pixel as^50^:

6 $$N = \frac{{(<I> - offset)^{2} }}{{\sigma^{2} - \sigma_{0}^{2} }} = \frac{\varepsilon n}{{\varepsilon + 1}}$$

7 $$B = \frac{{\sigma^{2} - \sigma_{0}^{2} }}{<I> - offset } = S\left( {\varepsilon + 1} \right)$$

B is the apparent brightness, N is the apparent number of molecules, <I> is the average signal intensity, σ^2^ is the variance of the signal, *offset* is the intensity offset, σ_0_^2^ is the readout noise variance of the analog detector, ε is the *true* molecular brightness and *n* is the *true* number of particles. An important parameter determined in the calibration of the instrument is the conversion factor S, which describes the properties of the microscope analog detection system converting from the photocurrent to photon counts^50^. For our instrument, the S factor was determined using the dark image method on a daily basis and varied between 6.8 and 7.3 (Supporting Fig. S8). To correct for changes in the fluorescence signals due to phenomena other than the changes in brightness such as photo-bleaching and slow cell movements, we applied a de-trending moving average filter of 7–10 frames to the stack of images before calculating the N&B parameters^65,66^.

To determine the oligomerization state of mEGFP-Drp1 in the cytosol of mammalian cells, the N&B method was calibrated by determining the brightness (B) of the non-oligomerizing mEGFP and assigning the calculated brightness to the monomer. This is achieved by expressing mEGFP in the same cell type and under the same conditions as for mEGFP-Drp1. The number of subunits in the oligomers of mEGFP-Drp1 is determined as:

8 $$\varepsilon_{norm.} = \frac{{\left( {B_{mEGFP - Drp1} - 1} \right)}}{{\left( {B_{mEGFP} - 1} \right)}}$$

ε_norm._ is the normalized molecular brightness, B_mEGFP-Drp1_ is the brightness of the cells expressing mEGFP-Drp1, and B_mEGFP_ is the brightness of the cells expressing mEGFP.

### Mitochondrial morphology

Images of live *wt* and *Drp1-null* MEFs expressing either mEGFP alone or mEGFP-Drp1 (488 nm excitation) were obtained using the Olympus FluoView FV1000 confocal microscope. Mitochondria were stained by the expression of the inner mitochondrial membrane-targeted fusion protein construct mCherry-Mito-7 (MITO, 543 nm excitation). Images obtained from the red and green channels were imported into the ImageJ software (version 1.52 h) for further processing. Brightness and contrast were enhanced using the software’s tools (brought up with the key combination Ctrl + Shift + C). The image dimensions were calibrated using the “set scale” form (Analyze/Set scale). Mitochondrial lengths were measured using the segmented line tool (to account for curved and other odd-shaped mitochondrial fragments) by drawing a line from end to end of each mitochondrial fragment, and then pressing the key combination “Ctrl + M”. At least five representative cells (~ 100 measurements per cell) were measured for each cell line. To obtain the number of puncta per μm length of mitochondria, each of the puncta observed in the measured fragments was counted and then divided by the mitochondrial length measured.

## Supplementary information

Supplementary file1
