# Supplementary material for: GFP fluorescence tagging alters dynamin-related protein 1 oligomerization dynamics and creates disassembly-refractory puncta to mediate mitochondrial fission
Source: Sci Rep. 2020 Sep 8;10:14777. doi: 10.1038/s41598-020-71655-x (PMC7479153; doi:10.1038/s41598-020-71655-x)
Supplement: Supplementary file 1 — Supplementary file1 [file 41598_2020_71655_MOESM1_ESM.pdf]

Supplementary Information

GFP fluorescence tagging alters dynamin-related protein 1 oligomerization dynamics and creates disassembly-refractory puncta to mediate mitochondrial fission

Felipe Montecinos-Franjola[1], Brianna L. Bauer[2], Jason A. Mears[2,3,4], Rajesh Ramachandran[1, 4,*]

Departments of Physiology and Biophysics[1], and Pharmacology[2],

[3]Center for Mitochondrial Diseases,

[4]Cleveland Center for Membrane and Structural Biology,

Case Western Reserve University School of Medicine,

Cleveland, OH 44106, USA

*Correspondence should be addressed to: rxr275@case.edu

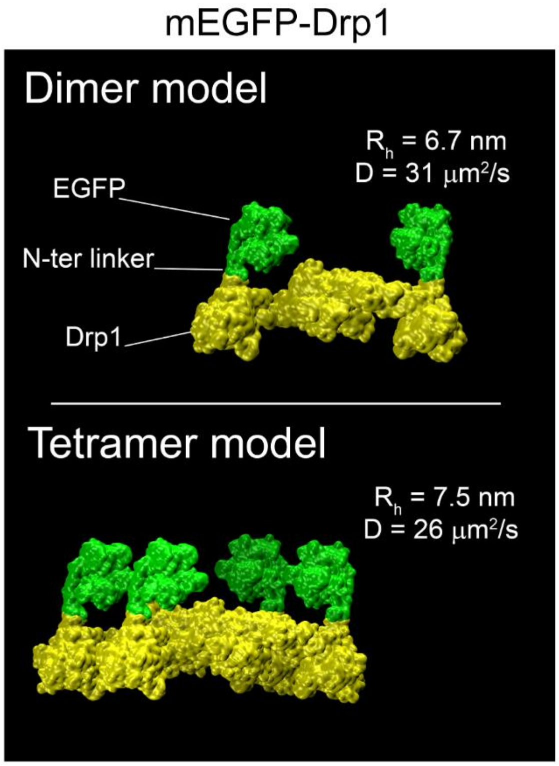

Predicted **D** obtained with Hydropro

Protein	PDB file	MW, kDa	D_1, $\mu m^2/s$
mEGFP	2Y0G	27	90
mEGFP-Drp1 dimer	Model	222	31
mEGFP-Drp1 tetramer	Model	444	26

Supporting Figure S1. Predicted structure and hydrodynamic properties of mEGFP-Drp1. (*Top*) I-TASSER[1]-predicted 3D structure of mEGFP-Drp1 (bottom-up view relative to Fig. 1A). The best-fit model of the mEGFP-Drp1 monomer was manually docked onto the Drp1ΔVD dimer structure[2] (PDB ID: 4BEJ) to produce the mEGFP-Drp1 dimer model. The corresponding tetramer model was generated by docking a pair of mEGFP-Drp1 dimers onto the dynamin tetramer structure[3] (PDB ID: 5A3F). All structural models were obtained using the VMD software[4]. (*Bottom*) The hydrodynamic radii and D of mEGFP-Drp1 in the dimer and tetramer forms were predicted from the above molecular models using the Hydropro software[5]. The corresponding information for mEGFP alone was generated from the available 3D structure (PDB ID: 2Y0G).

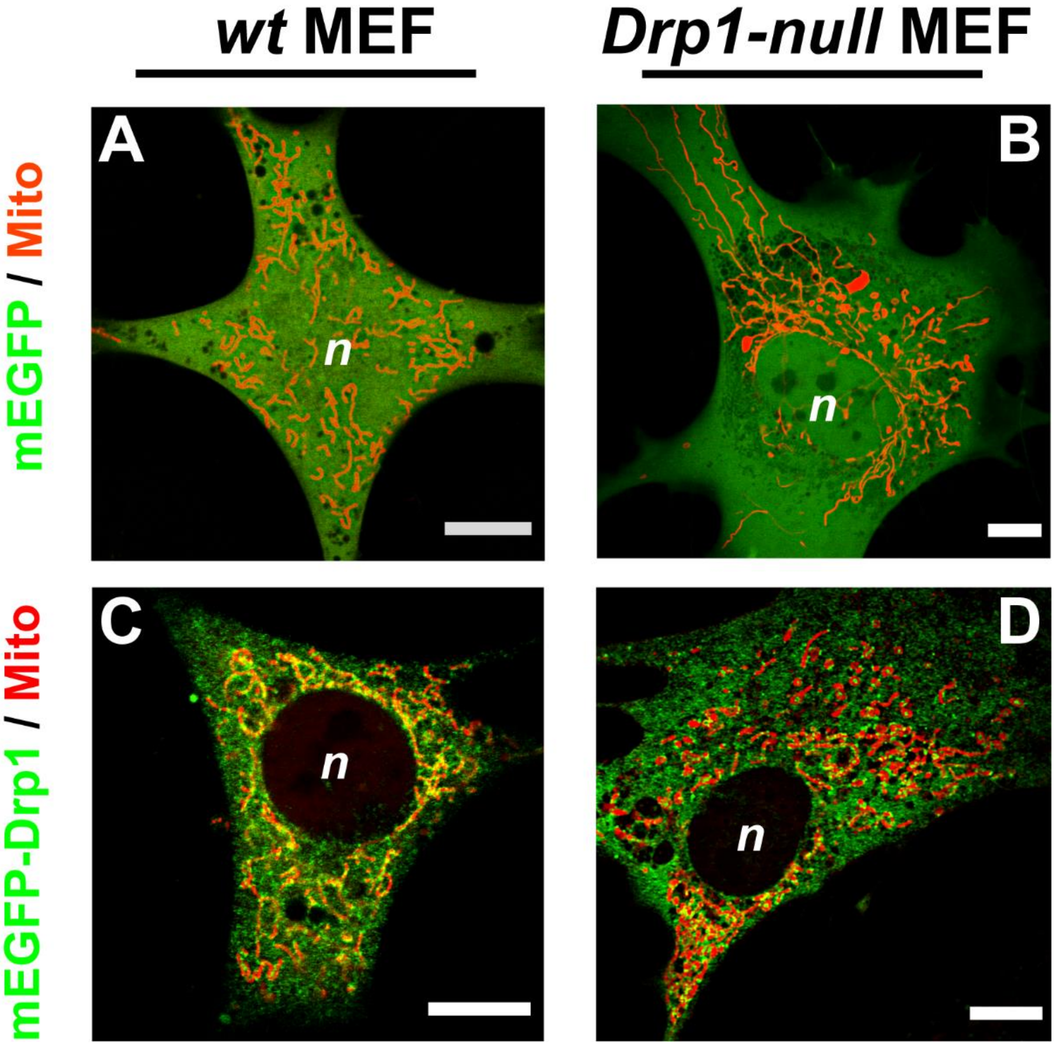

Supporting Figure S2. Confocal fluorescence images of *wt* and *Drp1-null* MEFs expressing either mEGFP alone (panels **A** and **B**) or mEGFP-Drp1 (panels **C** and **D**) (green). Mitochondria were visualized using mCherry-Mito-7 (red). Images were processed using the software platform Fiji[6]. Scale bar, 10 μm. n, nucleus.

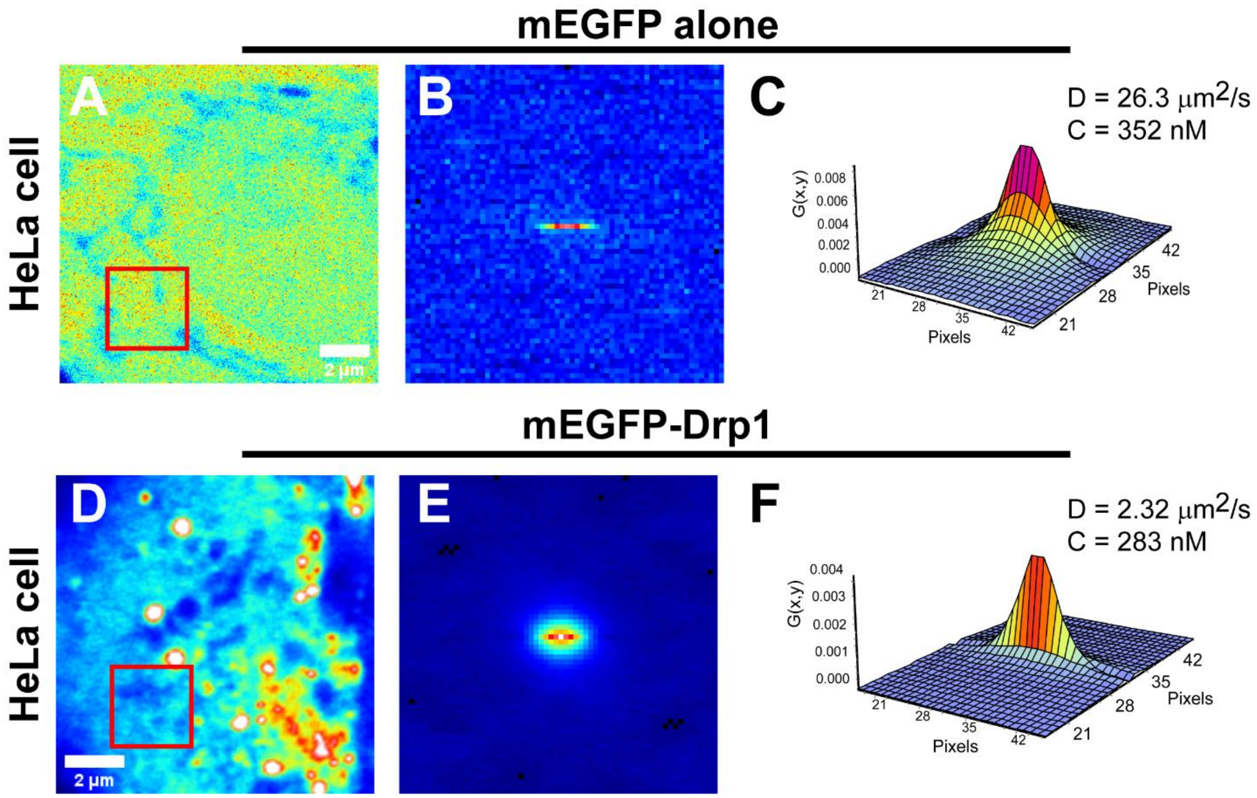

Supporting Figure S3. Determination of the D and concentration of mEGFP and mEGFP-Drp1 in the cytosol of a representative HeLa cell using RICS analysis. **(A**, **D**) Average intensity image of the cytosol used for RICS analysis. The red boxes enclose a 64×64 pixel area used for the analysis. Bright spots observed in panel D are large mEGFP-Drp1 puncta frequently observed in these cells. (**B**, **E**) Image of the spatial autocorrelation function obtained from the 64×64 pixel area (red boxes in panels **A** and **D**) indicated in the average intensity images. (**C**, **F**) Fits of the spatial autocorrelation function used for estimating D and the concentration of fluorescent molecules in the confocal volume. Scale bar, 2 μm. All images in this figure were obtained using the SimFCS 4 software available at https://www.lfd.uci.edu/globals/.

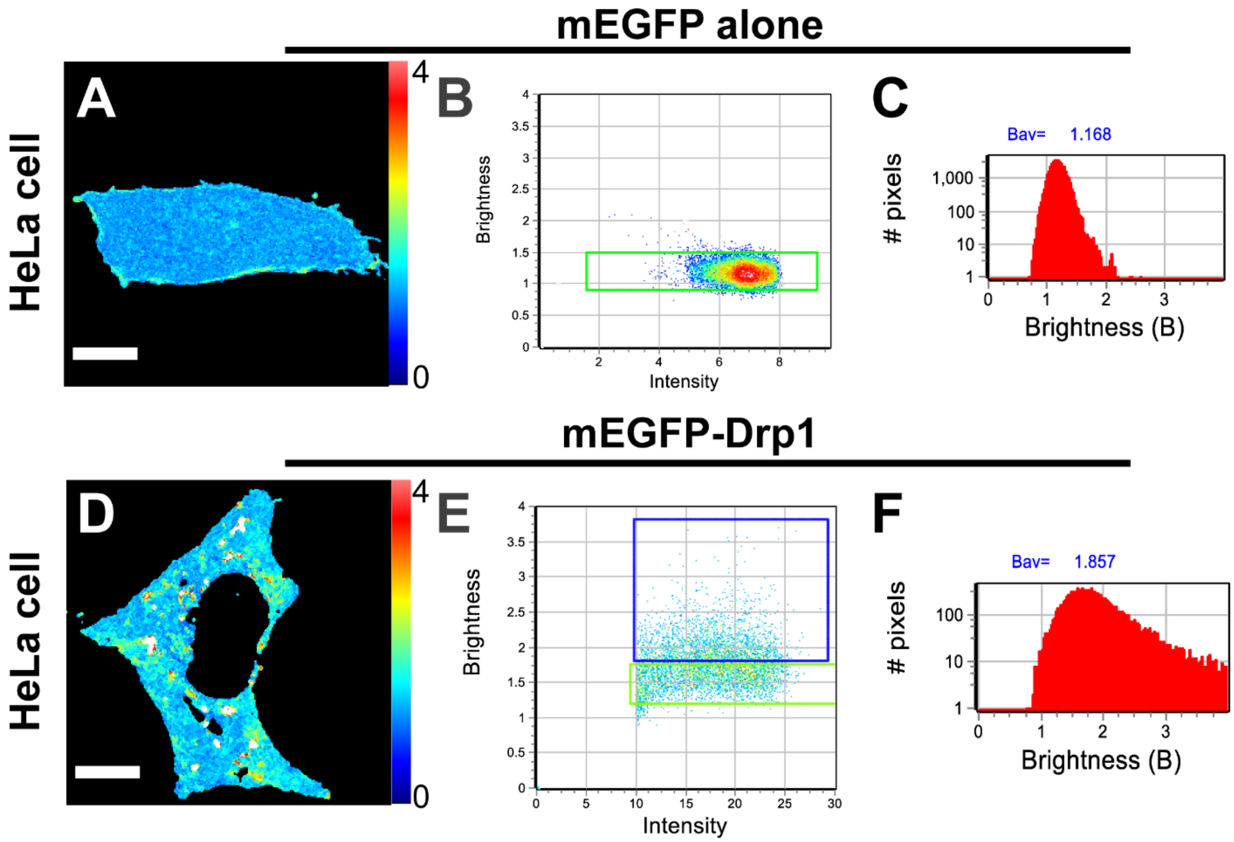

Supporting Figure S4. N&B analysis of mEGFP-Drp1 expressed in HeLa cells. (**A**, **D**) Distribution of background-subtracted B-values across the HeLa cell body. The pixel color corresponds to B-values scaled from 0 to 4 (100 frames were collected in each experiment, 50×50 μm each). (**B, E**) B-values of the collection of pixels from the images plotted against fluorescence intensity. The green boxes select pixels with average B = 1.17 for mEGFP and 1.55 for mEGFP-Drp1. (**C, F**) Histogram of B-values for mEGFP (panel **C**) shows a Gaussian distribution centered at B = 1.17 ± 0.10, whereas that of mEGFP-Drp1 (panel **F**) shows a broadening towards higher values with a maximum centered at B = 1.79 ± 0.56 (fitted to a Gaussian distribution). HeLa cells expressed higher levels of mEGFP-Drp1 relative to MEFs resulting in larger puncta distributed across the cytosol. These pixels showed saturation (white pixels in panel **D**) in the B-maps due to the high fluorescence intensities recorded. Scale bar, 10 μm. All images in this figure were obtained using the SimFCS 4 software available at https://www.lfd.uci.edu/globals/.

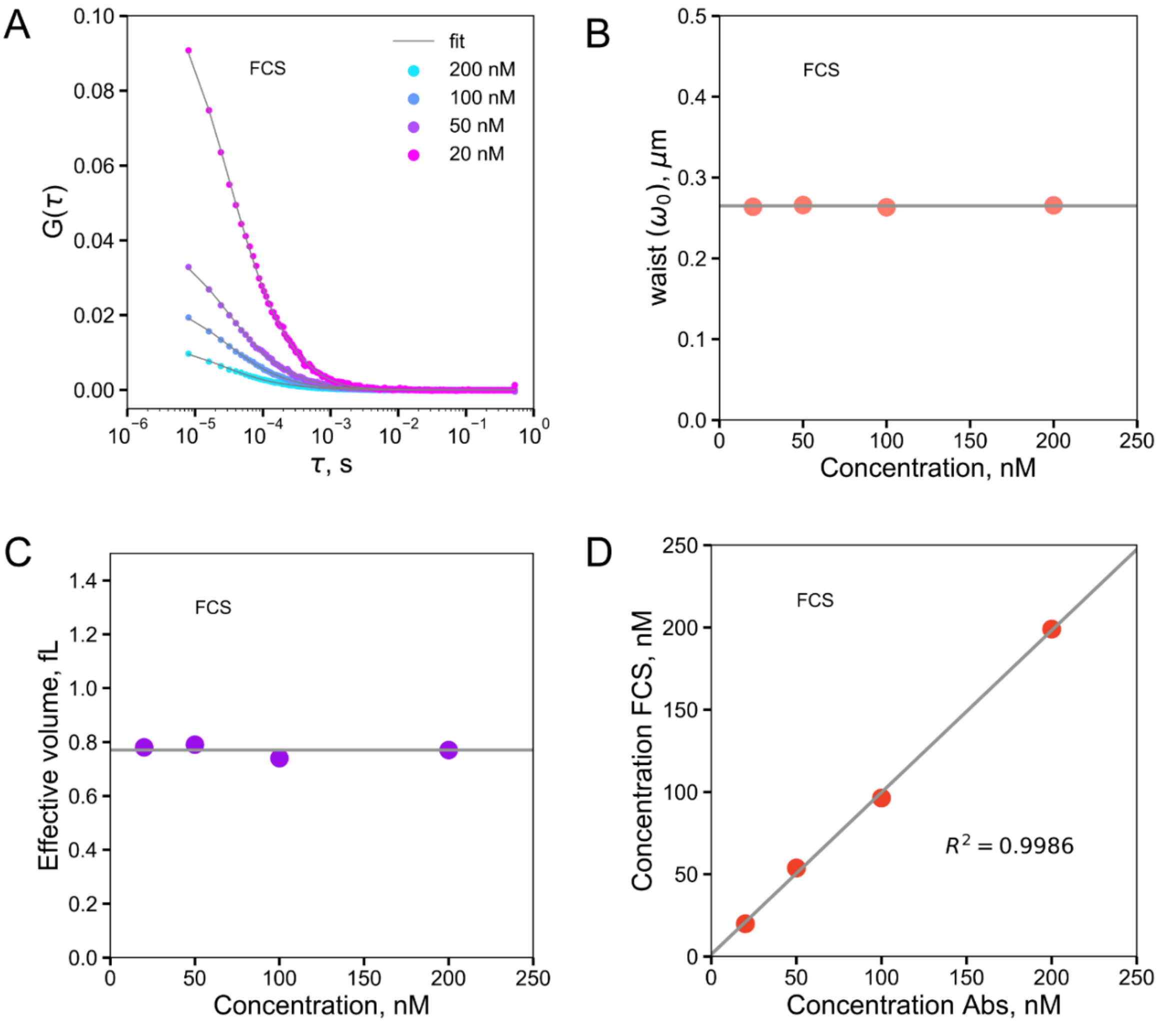

Supporting Figure S5. Calibration of the confocal volume with the reference dye AF488 for single-point FCS measurements. **(A**) Recovered autocorrelation curves for AF488 in the 20 – 200 nM concentration range. D_{AF488} = 435 $\mu m^2/s$ was used[7]. The experimental data were fit to a single diffusing component (solid lines). (**B)** Best-fit radial beam waist (ω_r) for each of the AF488 concentrations tested. A constant average value of ω_r = 0.265 ± 0.001 μm was obtained in this range of concentrations. The structural parameter of the confocal volume ω_z/ω_r was assumed to be equal to 3[ref.8]. (**C)** The effective observation volume was calculated using the equation $V_{eff} = 1/(G(0) \times N_A \times C$ where G(0) is the amplitude of the autocorrelation function, N_A is the Avogadro number and C is the concentration used in the experiment. The calculated effective observation volume of V_{eff} = 0.77 ± 0.02 fL was constant in this range of concentrations, and was assumed to be related to the 3D Gaussian confocal volume $V_{conf} = (1/2)^{3/2} \times V_{eff}$. (**D)** Estimation of the concentration of fluorescent molecules in the confocal volume determined using the number of molecules obtained from the autocorrelation functions shown in panel **A**. Excellent agreement was found between loading dye concentration determined by absorbance and the concentration estimated using FCS indicating a precise calibration of the confocal volume. The number of molecules was calculated using $N = \gamma/G(0)$, where γ = 0.3536 for a 3D Gaussian confocal volume. The plots were prepared using matplotlib v3.2.2 available at https://matplotlib.org/3.2.2/api/_as_gen/matplotlib.pyplot.boxplot.html.

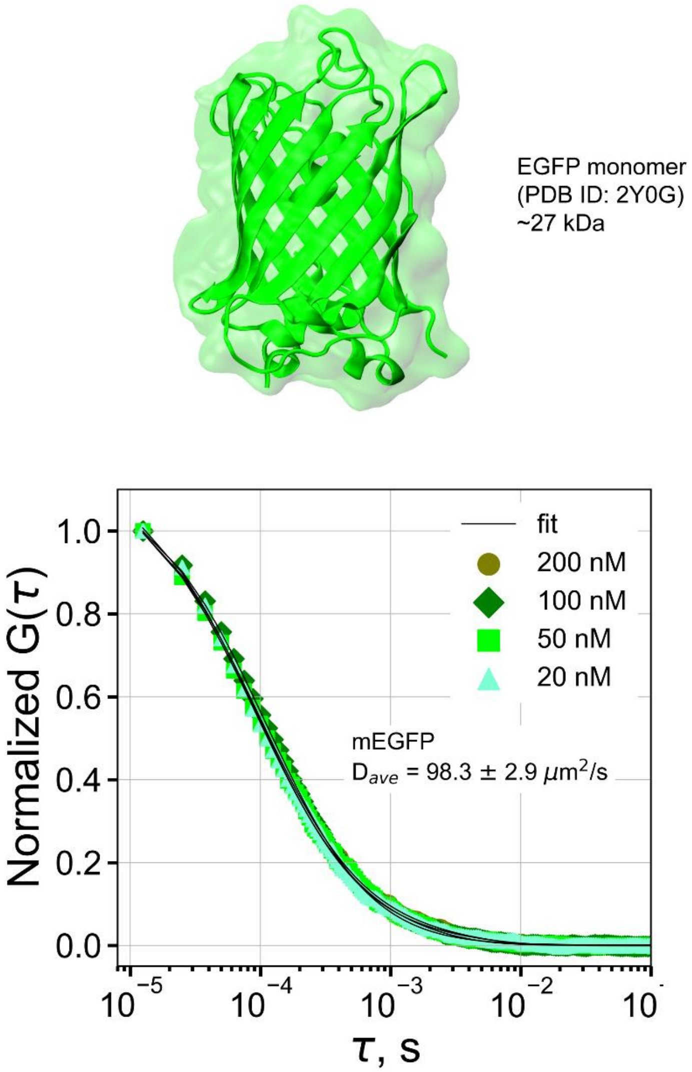

Supporting Figure S6. Concentration-independent monomeric state of mEGFP. (*Top*) Crystal structure and approximate size of the EGFP monomer (PDB ID: 2Y0G). (*Bottom*) Purified mEGFP was analyzed by single-point FCS in the 20 – 200 nM concentration range and D was determined from the fits of the autocorrelation curves obtained (solid lines). D was invariant (<5% change) in this range of concentrations. D recovered for mEGFP alone (D_{ave}= 98.3 ± 2.9 $\mu m^2/s$) is consistent with reported values that range between 90 and 100 $\mu m^2/s$[7,9,10]. The structural model of EGFP was prepared using VMD v1.9.3[4] available at http://www.ks.uiuc.edu/Research/vmd/. The autocorrelation plot was prepared using matplotlib v3.2.2 available at https://matplotlib.org/3.2.2/api/_as_gen/matplotlib.pyplot.boxplot.html.

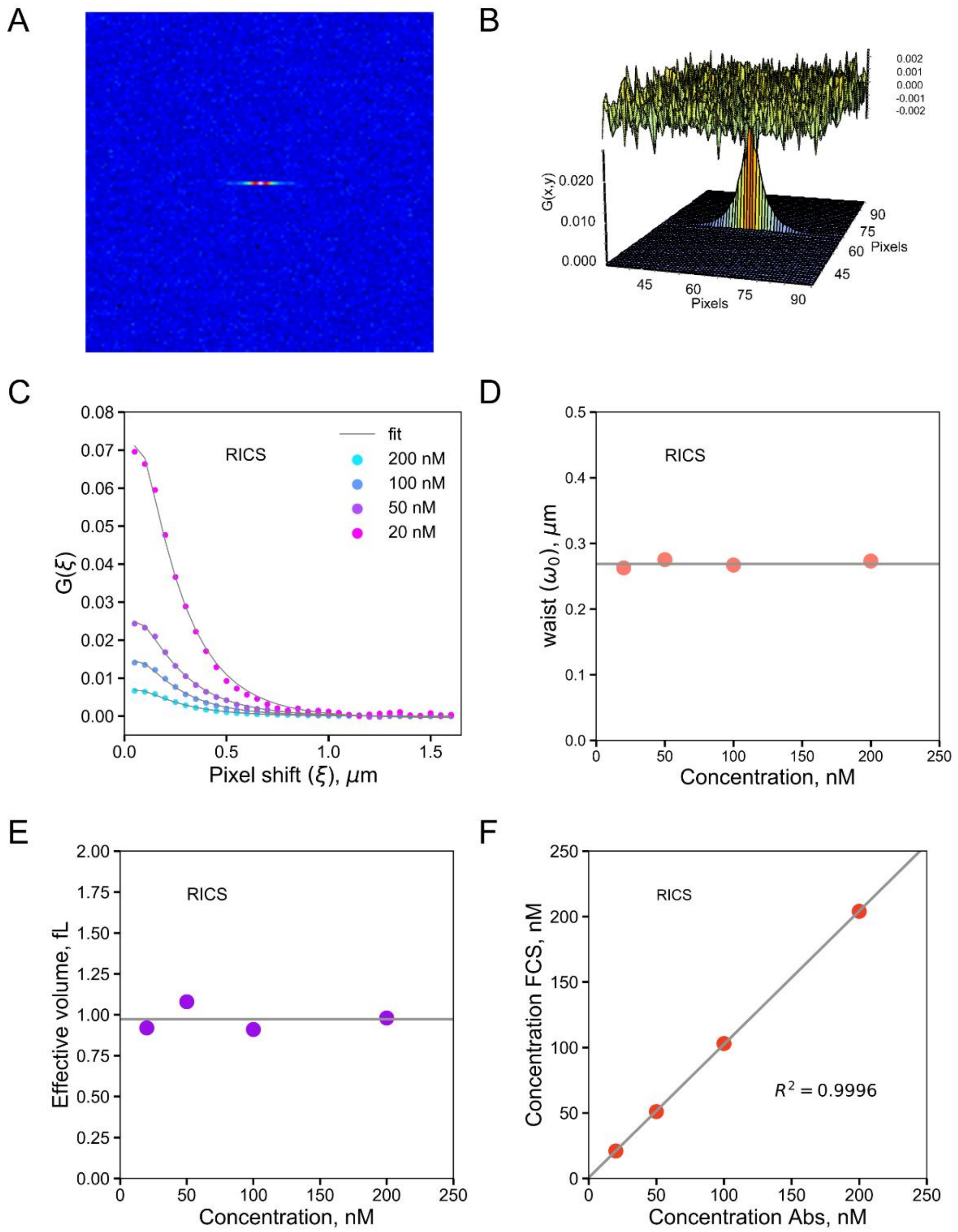

Supporting Figure S7. Calibration of the confocal volume with the reference dye AF488 for RICS measurements**. (A**) Spatial autocorrelation function (ACF) obtained by RICS analysis of a solution of AF488 at 100 nM using a stack of 100 frames (pixel size = 0.05 μm, pixel time = 8 μs, frame size = 256×256 pixels). The spatial ACF appears elongated in the horizontal axis due to fast diffusion of the fluorescent molecules. (**B**) RICS surface fit of the spatial ACF shown in panel **A** (64×64 pixel area). The residuals of the fits is shown at the top. (**C**) Recovered horizontal ACF of AF488 in the 20 – 200 nM concentration range. D_{AF488} = 435 $\mu m^2/s$ was used[7]. The obtained ACF was fit with a single diffusing component (solid lines). (**D)** Best-fit radial beam waist (ω_r) for each of the AF488 concentrations tested. An average constant value of ω_r = 0.269 ± 0.005 μm was obtained for this range of concentrations. The structural parameter of the confocal volume, which

is the ratio of axial and radial beam waists (ω_z/ω_r), was assumed to be equal to 3[ref.8]. (**E)** The effective observation volume was calculated using the equation $V_{eff} = 1/(G(0)\times N_A\times C)$, where G(0) is the amplitude of the autocorrelation function, N_A is the Avogadro number and C is the concentration used in the experiment. A constant $V_{eff} = 0.97 \pm 0.08$ fL was estimated for this range of concentrations, and was assumed to be related to the 3D Gaussian confocal volume by $V_{conf} = (1/2)^{3/2}\times V_{eff}$. (**F**) Estimation of the concentration of fluorescent molecules in the confocal volume determined from the number of molecules N obtained from the best-fit G(0) of the spatial ACF shown in panel **C**. Good agreement between the loading dye concentration determined by absorbance and the concentration estimated from the RICS analyses was found indicating a precise calibration of the confocal volume. The number of molecules was calculated using $N = \gamma/G(0)$, where $\gamma = 0.3536$ for the 3D Gaussian confocal volume. The spatial ACF and the fit plot in panel **B** were obtained using the SimFCS 4 software available at https://www.lfd.uci.edu/globals/. Plots in panels **C-F** were prepared using matplotlib v3.2.2 available at https://matplotlib.org/3.2.2/api/_as_gen/matplotlib.pyplot.boxplot.html.

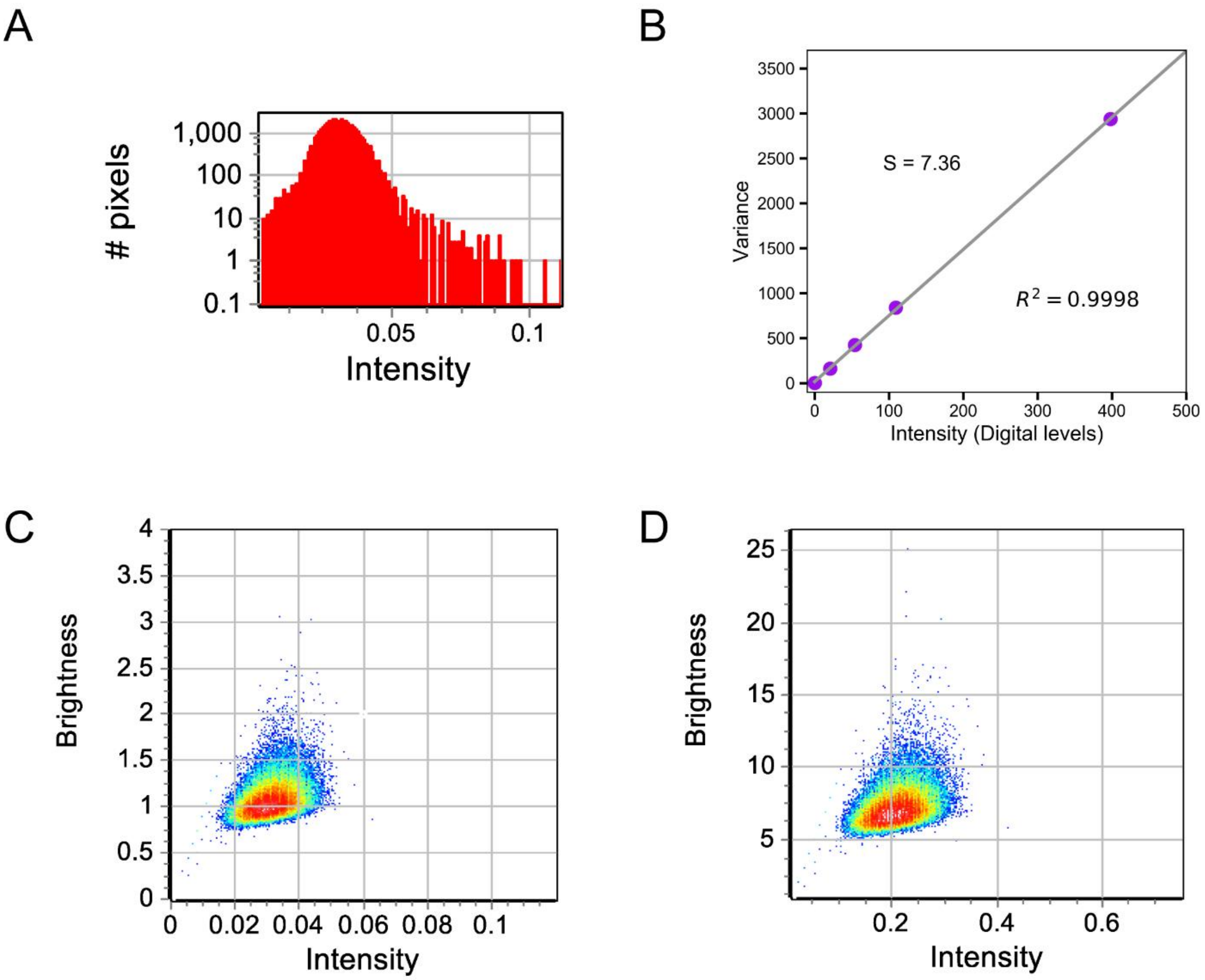

Supporting Figure S8. Calibration of the detector parameters for estimation of molecular brightness and particle number. (**A**) Distribution of digital levels for the dark image (with the laser turned off) of the PMT used for all experiments. The pixel dwell time was 12.5 μs, and 100 frames were collected. The center of the Gaussian part of the dark image histogram is the detector offset (offset = 0.033) and the width is the readout noise (σ_0 = 0.0007). (**B)** Determination of the conversion factor S. For these measurements, an empty microscopy dish was illuminated with varying (increasing) laser power and 100 frames were collected. For each acquisition, the signal variance and average intensity were determined and plotted. The slope of this plot is equal to the conversion factor, S = 7.36, which was constant for the range of intensities of the samples used in these experiments. (**C**, **D**) Values of brightness versus intensity for each pixel of the dark image, after (panel **C**) and before (panel **D**) application of the conversion factor S. As expected, the signal obtained from an immobile sample (empty dish) has a brightness equal to 1 after applying the conversion factor S (panel **C**). The histogram and brightness plots in panels **A**, **C**, and **D** were obtained using the SimFCS 4 software available at https://www.lfd.uci.edu/globals/. Plot in panel **B** was prepared using matplotlib v3.2.2 available at https://matplotlib.org/3.2.2/api/_as_gen/matplotlib.pyplot.boxplot.html.

References

1. Roy, A., Kucukural, A. & Zhang, Y. I-TASSER: a unified platform for automated protein structure and function prediction. *Nat Protoc* **5**, 725-38 (2010).
2. Frohlich, C. et al. Structural insights into oligomerization and mitochondrial remodelling of dynamin 1-like protein. *EMBO J* **32**, 1280-92 (2013).
3. Reubold, T.F. et al. Crystal structure of the dynamin tetramer. *Nature* **525**, 404-8 (2015).
4. Humphrey, W., Dalke, A. & Schulten, K. VMD: visual molecular dynamics. *J Mol Graph* **14**, 33-8, 27-8 (1996).
5. Garcia De La Torre, J., Huertas, M.L. & Carrasco, B. Calculation of hydrodynamic properties of globular proteins from their atomic-level structure. *Biophys J* **78**, 719-30 (2000).
6. Schindelin, J. et al. Fiji: an open-source platform for biological-image analysis. *Nat Methods* **9**, 676-82 (2012).
7. Petrasek, Z. & Schwille, P. Precise measurement of diffusion coefficients using scanning fluorescence correlation spectroscopy. *Biophys J* **94**, 1437-48 (2008).
8. Digman, M.A. et al. Measuring fast dynamics in solutions and cells with a laser scanning microscope. *Biophys J* **89**, 1317-27 (2005).
9. Moens, P.D., Gratton, E. & Salvemini, I.L. Fluorescence correlation spectroscopy, raster image correlation spectroscopy, and number and brightness on a commercial confocal laser scanning microscope with analog detectors (Nikon C1). *Microsc Res Tech* **74**, 377-88 (2011).
10. Jameson, D.M., Ross, J.A. & Albanesi, J.P. Fluorescence fluctuation spectroscopy: ushering in a new age of enlightenment for cellular dynamics. *Biophys Rev* **1**, 105-118 (2009).
